# Exploring Canadian Echinoderm Diversity through DNA Barcodes

**DOI:** 10.1371/journal.pone.0166118

**Published:** 2016-11-21

**Authors:** Kara K. S. Layton, Erin A. Corstorphine, Paul D. N. Hebert

**Affiliations:** Centre for Biodiversity Genomics, Biodiversity Institute of Ontario, University of Guelph, Guelph, ON, N1G 2W1, Canada; Universita degli Studi della Tuscia, ITALY

## Abstract

DNA barcoding has proven an effective tool for species identification in varied groups of marine invertebrates including crustaceans, molluscs, polychaetes and echinoderms. In this study, we further validate its utility by analyzing almost half of the 300 species of Echinodermata known from Canadian waters. COI sequences from 999 specimens were assigned to 145 BINs. In most cases, species discrimination was straightforward due to the large difference (25-fold) between mean intra- (0.48%) and inter- (12.0%) specific divergence. Six species were flagged for further taxonomic investigation because specimens assigned to them fell into two or three discrete sequence clusters. The potential influence of larval dispersal capacity and glacial events on patterns of genetic diversity is discussed for 19 trans-oceanic species. Although additional research is needed to clarify biogeographic patterns and resolve taxonomic questions, this study represents an important step in the assembly of a DNA barcode library for all Canadian echinoderms, a valuable resource for future biosurveillance programs.

## Introduction

DNA barcoding employs sequence variation in a 648 bp region of the mitochondrial cytochrome *c* oxidase subunit 1 (COI) gene as a tool for specimen identification and species discovery [1]. Its effectiveness has been shown in birds [2,3], bats [4], fishes [5–7], insects [8,9] and several marine invertebrate groups, including stomatopods [10], molluscs [11,12], crustaceans [13], and echinoderms [14]. The routine occurrence of a barcode gap, where interspecific divergence is much greater than intraspecific divergence, underpins its strong performance in taxonomic assignments versus the complexities which often complicate morphological approaches. For example, a survey of 15 marine phyla reported that one third of specimens could not be assigned to a species through morphological analysis [15] because of factors such as damage to diagnostic characters during collection. Immature stages are often particularly challenging to identify beyond a family via morphology, making it difficult to estimate the timing and duration of larval dispersal [16]. The use of DNA barcoding for species identification solves many of the problems associated with the application of morphology-based approaches. It has, for example, proven highly effective in identifying immature stages of fishes [17] and varied lineages of marine invertebrates [10,16,18]. It is particularly useful when a comprehensive barcode reference library is available. Moreover, divergence thresholds are a powerful tool for revealing newly encountered species, albeit they can overlook recently diverged species [10,19].

Although their phylogenetic relationships, developmental patterns, and reproductive biology have been well investigated, Canadian echinoderms have seen little genetic analysis. Three studies have examined introgression along secondary contact zones [20–22], while another investigated a cryptic species complex [23]. A further investigation documented patterns of mtDNA divergence in four trans-Arctic sea urchin species [24] while another used COI sequences to assess phylogeographic patterns in Arctic marine invertebrates, including several echinoderms from this region of North America [25]. While little information is available on patterns of COI sequence divergence in Canadian echinoderms, studies on this phylum in other regions have reported high interspecific divergences (2.5–24.2%) and low intraspecific distances (<1%) at COI [14,26,27]. Although these values are similar to those in other marine invertebrates [11,13,28] and fishes [6], low divergences were detected between some sibling species of echinoderms. For example, divergences of 1.1–1.2% for COI were observed between sister taxa of sea stars [29], while interspecific divergences of 2–3% were noted in closely-related echinoid species [30].

The variable climatic and hydrographic conditions of Canada’s oceans during the late Cenozoic [31] means that its marine species have complex histories of range expansion and fragmentation. For example, the opening of the Bering Strait during the mid-Pliocene (3.5 Mya), coupled with ice-free Arctic waters [32], allowed extensive migration between the north Pacific and Arctic-Atlantic oceans [33,34]. Subsequent Pleistocene glaciations eradicated much of the fauna in the Arctic and northwest Atlantic where conditions were more severe than in the Pacific. Differing histories of re-colonization from Pacific and Atlantic refugia following deglaciation approximately 14,000 years ago [32] is reflected in varied levels of population structure, ranging from closely-related species complexes (*Macoma*, [35]; *Mallotus*, [36]) to widespread species with little genetic divergence (*Strongylocentrotus*, [24,37]).

This study begins the construction of a DNA barcode reference library for Canadian echinoderms, but since several of these taxa also occur in polar and temperate regions across the northern hemisphere, the library has broader utility. Approximately 300 species of echinoderms are known from Canada's oceans with two-thirds (217) restricted to the NE Pacific [38–40]. The present study tests the efficacy of DNA barcoding and examines phylogeographic patterns resulting from Canada's complex glacial history. By focusing on Canadian echinoderms, the size of the fauna (300 species) makes it feasible to develop a comprehensive reference library. Moreover, the circumboreal distributions of several species provide an opportunity to investigate patterns of intraspecific divergence at a large geographic scale.

## Methods

### Ethics Statement

Fieldwork in Churchill, Manitoba was conducted under permits issued by Manitoba Conservation Wildlife and Ecosystem Protection to the Churchill Northern Studies Centre (CNSC) for research in the Churchill Wildlife Management Area. Collections in British Columbia, Labrador, and New Brunswick were conducted under licenses from Fisheries and Oceans Canada. No specific permits were required for other collection activities as they were not conducted on privately owned or protected land. No field studies involved the collection of endangered or protected species. A list of BOLD sample IDs, process IDs, GenBank accession numbers, and the institution storing each specimen in this study is available in S1 Table.

### Specimen collection and taxonomy

**Fig 1:**
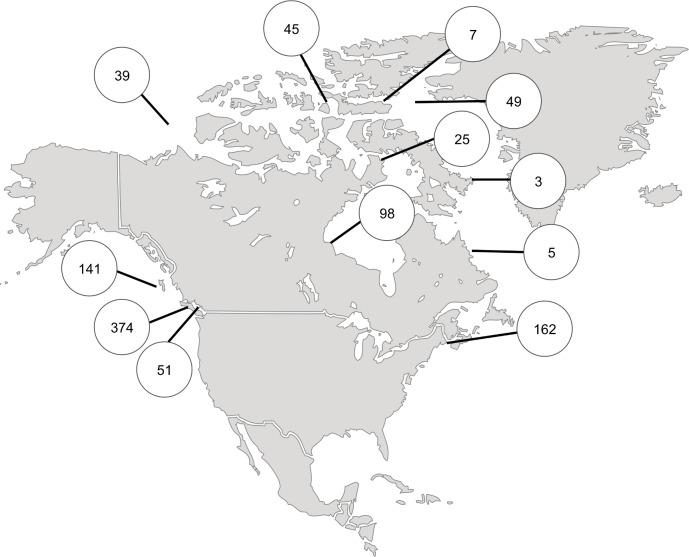
Sampling map. Collection localities and sample sizes for 999 specimens that generated a COI sequence in this study.

Tissue samples were obtained from 1285 specimens in the collections of the Centre for Biodiversity Genomics (CBG) and the Royal British Columbia Museum (RBCM). These samples included 316 specimens representing 78 species from trawl collections made by the RBCM between 2000 and 2006 at depths > 200m at various localities along the coast of British Columbia (Fig 1). Another 252 specimens were obtained by SCUBA and dredging between 1995 and 2002 from Queen Charlotte Sound and Nunavut (Fig 1) while 589 specimens were collected by SCUBA, by dredging and by hand from sites near Baffin Island, British Columbia, Labrador, Manitoba, and New Brunswick between 2007 and 2014 (Fig 1). A final 128 specimens were gathered by trawls at depths < 700m from the Beaufort Sea, Baffin Bay and the Labrador Sea in 2010 as part of the Canadian Healthy Oceans Network (Fig 1). Scientific names follow the World Register of Marine Species (WoRMS, http://www.marinespecies.org). All RBCM specimens were identified by Philip Lambert, an echinoderm specialist. All CBG specimens were identified using regional taxonomic keys (British Columbia: [38–41]; Atlantic and Arctic Canada: [42]). When a species-level identification could not be made, an interim name was assigned that coupled a genus-level identification with a Barcode Index Number (BIN) designation [43]. All specimens are stored as vouchers in 95% ethanol or frozen at -20°C in collections at the RBCM or the CBG (S1 Table). Specimen and sequence data are available in the dataset DS-COIECH dx.doi.org/10.5883/DS-COIECH on BOLD, the Barcode of Life Data Systems [44]. All sequences have also been deposited in GenBank under the following accession numbers: GU670162-65; GU670167-81; GU670187-94; HM400305-69; HM400539; HM405487; HM405870-914; HM473811-74; HM473876-89; HM473902-57; HM542062-420; HM542908-3073; JF891304-16; JN295388; JN314244; KU495734-918.

### DNA extraction, COI amplification, and sequencing

A DNA extract was prepared from the tube feet or gonadal tissue of each specimen. Tissue was placed into 96-well plates containing modified CTAB lysis buffer [45] and proteinase K (20mg/mL). Samples were incubated at 56°C for 18–24 hours before extraction using a 3um filter plate following a standard manual protocol [45]. Extracts were re-suspended in 40 μL of molecular grade water. COI was amplified using several primer combinations with products being 652bp, 658bp or 841bp in length depending on the primer set (Table 1). Polymerase chain reactions (PCRs) were carried out in 12.5 μL reaction volumes containing: 6.25 μL 10% trehalose, 2 μL molecular grade water, 1.25 μL 10X PCR Platinum Taq buffer, 0.625 μL MgCl_2_ (50 mM), 0.125 μL of each primer (10 mM), 0.0625 μL dNTPs (10 mM), 0.06 μL of Platinum Taq polymerase and 2 μL of DNA template (20–60 ng) using a thermocycling profile of one cycle at 94°C for 1 min, five cycles at 94°C for 40 s, 45°C for 40 s, and 72°C for 1 min, 35 cycles of 94°C for 40 s, 51°C for 40 s and 72°C for 1 min, and a further extension period of 72°C for 5 min. PCR products were visualized on pre-cast 2% agarose gels (E-gel 96, Invitrogen) and PCR products with single, bright bands were selected for bidirectional sequencing using BigDye version 3.1 on a 3730XL DNA Analyser (Applied Biosystems). Cycle-sequencing reactions incorporated the same primers as those used to generate the selected PCR product. Sequences from specimens lacking a species designation were analyzed with both the BOLD identification engine and BLAST in GenBank [46] to determine if sequence matches existed. Congruence between identifications based on sequence data and morphological characters was confirmed prior to assigning names to unidentified specimens.

**Table 1:** Primers used in this study.

Primer name	Primer sequence (5’-3’)	Length of amplicon (bp)	References
EchinoF1	TTTCAACTAATCATAAGGACATTGG	841	[14]
COIeR1	GCTCGTGTRTCTACRTCCAT		[26]
EchinoF1	TTTCAACTAATCATAAGGACATTGG	658	[14]
HCO2198	TAAACTTCAGGGTGACCAAAAAATCA		[47]
LCOech1aF1	TTTTTTCTACTAAACACAAGGATATTGG	658	This study
HCO2198	TAAACTTCAGGGTGACCAAAAAATCA		[47]
COIeF1	ATAATGATAGGAGGRTTTGG	652	[26]
COIeR1	GCTCGTGTRTCTACRTCCAT		[26]

Four primer sets used in this study with their amplicon length and references for both forward and reverse primers, respectively.

### Data analysis

All sequences were edited manually using Sequencher 4.8 and aligned by eye in MEGA 6.0 [48]. As most sequences were barcode compliant (N = 987), they received a Barcode Index Number (BIN), aiding in species delimitation [43]. Maximum-likelihood trees were constructed with 1000 bootstrap replicates and either an HKY+I model in MEGA 6.0 [48] or with a GTR+G+I model in RAxML v8 [49]. Models with the highest AIC values generated by jModelTest v2 [50,51] were selected as most appropriate. Pairwise estimates of intra- and interspecific sequence divergence were calculated using the K2P distance model [52] and the Distance Summary tools on BOLD [44]. The presence of a barcode gap was analysed by plotting maximum intraspecific divergence against Nearest-Neighbor distance. All species with an intraspecific divergence >2% were flagged for further investigation. Maximum and mean intraspecific divergences were plotted against the number of individuals sampled within a species and regression analysis was carried out in RStudio using the Picante and VEGAN packages to determine the significance of this relationship [53,54].

## Results

### Sequence recovery

COI sequences were recovered from 999 specimens representing 141 species, 77 genera and 43 families for a success rate of 78% (999/1285). Sequences ranged in length from 347bp to 658bp but 95% were over 600bp. COI sequences could not be recovered from 18 species of deep-sea echinoderms from the RBCM collection despite their young age (2–10 years). Nuclear pseudogenes of mitochondrial origin (NUMTs, [55]) were occasionally encountered along with authentic COI, but most were <180bp so full sequences could be recovered following deletion of the NUMT region in the initial segment of each forward and reverse read.

### Taxonomic issues

The 141 presumptive species included 118 taxa with species-level identifications based on morphological study. The other 23 species could only be placed to a genus, but were assigned interim species identifications based on their BIN membership (e.g. *Ceramaster* sp. *AAI7443*). Many of these 23 taxa may represent undescribed species or taxa previously undocumented from Canada. For instance, two specimens of sea urchin (*Strongylocentrotus* sp. *AAA9523*) collected in arctic Canada did not match any species known from this region. Several characters commonly used to identify urchins, such as number of pore pairs and spine wedges, overlap in the other two Canadian members of this genus (*S*. *droebachiensis*, *S*. *pallidus*) and were similarly undiagnostic in this new taxon. However, it was distinguished morphologically by the deep purple colouration of its test, tube feet and spines. In addition, the density and size of spines on its test were reduced in comparison with other *Strongylocentrotus* specimens from Canada. Certain cases where there was initial discordance between barcode results and taxonomic assignments were resolved when taxonomic reanalysis led to their placement in the species corresponding with the barcode data. Many of these cases involved juveniles or genera where two species have subtle morphological differences.

### COI variation

**Fig 2:**
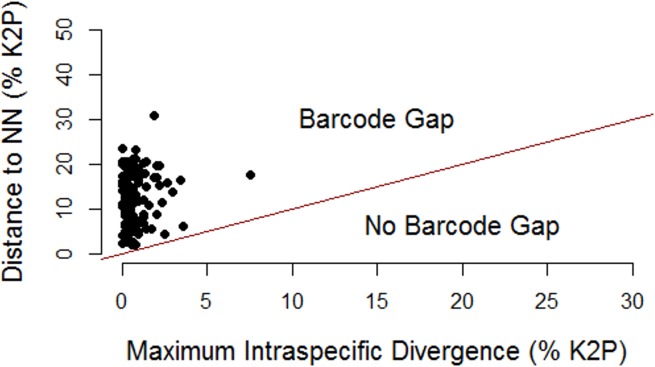
Barcode gap. Nearest-Neighbor distances (% K2P) plotted against maximum intraspecific divergences (% K2P) for 113 taxa with two or more individuals. All taxa show a barcode gap.

**Fig 3:**
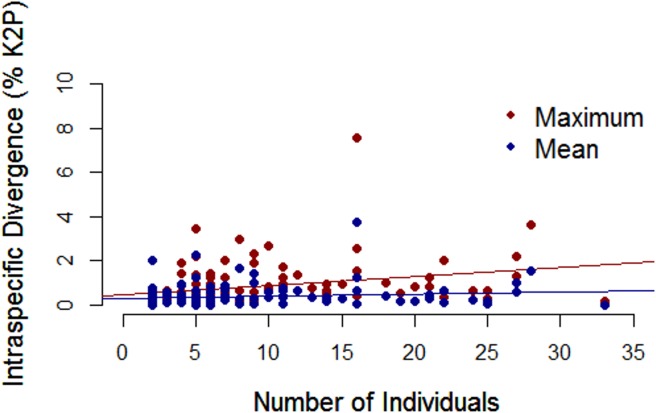
Relationship between COI distance and sample size. Maximum and mean intraspecific divergences (% K2P) plotted against the number of individuals sampled for 113 species. The regression between maximum intraspecific divergence and the number of individuals sampled is significant (R^2^ = 0.089, p<0.01).

**Fig 4:**
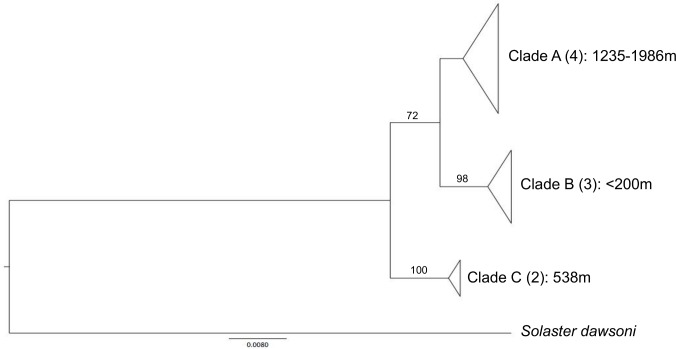
Depth partitioning in *Lophaster furcilliger*. Maximum-likelihood tree (HKY+I) for specimens of *Lophaster furcilliger* from British Columbia. Groups A, B and C are partitioned by depth and sequence number is presented in brackets for each clade. Scale bar represents percent sequence divergence.

An average of seven individuals were sequenced per species (range of 1 to 33) with 113 taxa represented by multiple specimens and 28 taxa by singletons. Mean intraspecific distance was 0.48% (range 0.0–7.6%) while mean interspecific divergence was 25-fold greater at 12.0% (range 2.0–26.2%). S2 Table presents mean and maximum intraspecific distances for each of the 113 morphospecies represented by two or more specimens. A barcode gap was present for all taxa (Fig 2) although 12 species had maximum intraspecific divergences greater than 2%. Values of maximum intraspecific divergence showed a significant increase with the number of individuals sampled, but the coefficient of determination was low (Fig 3). Four morphospecies were not assigned a BIN because their sequence length was less than 500bp, but the other 137 were assigned to 145 BINs reflecting the fact that six species (*Gorgonocephalus arcticus*, *Leptasterias hexactis*, *Leptosynapta clarki*, *Lophaster furcilliger*, *Ophiura sarsii*, *Pteraster militaris*) included two or three clusters, each assigned to a different BIN. S3 Table reports the number of sequences and locality information for each BIN. A ML tree for one of these species, *L*. *furcilliger*, showed genetic partitioning associated with water depth with specimens in cluster A collected from 1200–2000m while those in cluster B were found at < 200m and cluster C at 500m (Fig 4). When these six taxa with multiple BINs (which may represent cryptic species) were removed from the analysis, the mean intraspecific divergence dropped to 0.32%. Mean Nearest-Neighbor distances were lowest between *S*. *droebachiensis* and *S*. sp. *AAA9523* (2.0%).

**Fig 5:**
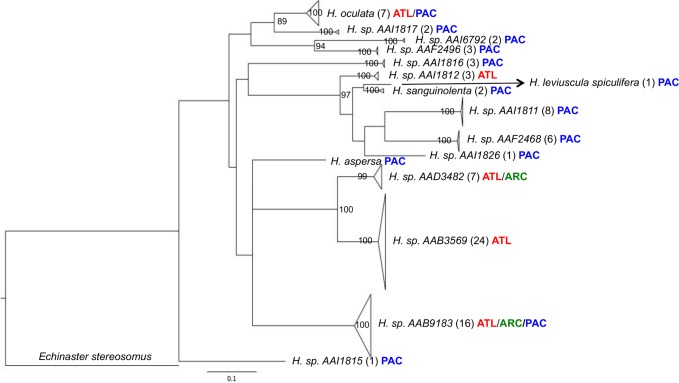
Deep divergences at COI within *Henricia*. Maximum-likelihood tree (GTR+G+I) for 16 putative species of *Henricia*. Locality information is provided for each lineage (Atl = Atlantic, Arc = Arctic, Pac = Pacific) and sequence number is presented in brackets for each clade. Scale bar represents percent sequence divergence.

Some specimens of *Henricia* could not be assigned to a species because morphological differences among species in this genus are so conserved. However, the 87 sequences for this genus formed 16 clusters with relatively high interspecific distances (mean 13.7%; range 2.6–18.3%) and low intra-group divergences (mean 0.33%; range 0–1.6%) (Fig 5). Among the 12 clusters represented by multiple specimens, seven were only found in the Pacific, two in the Atlantic, one in both the Pacific and Atlantic, one in both the Arctic and Atlantic, and another in all three oceans.

### Divergence across oceans

**Fig 6:**
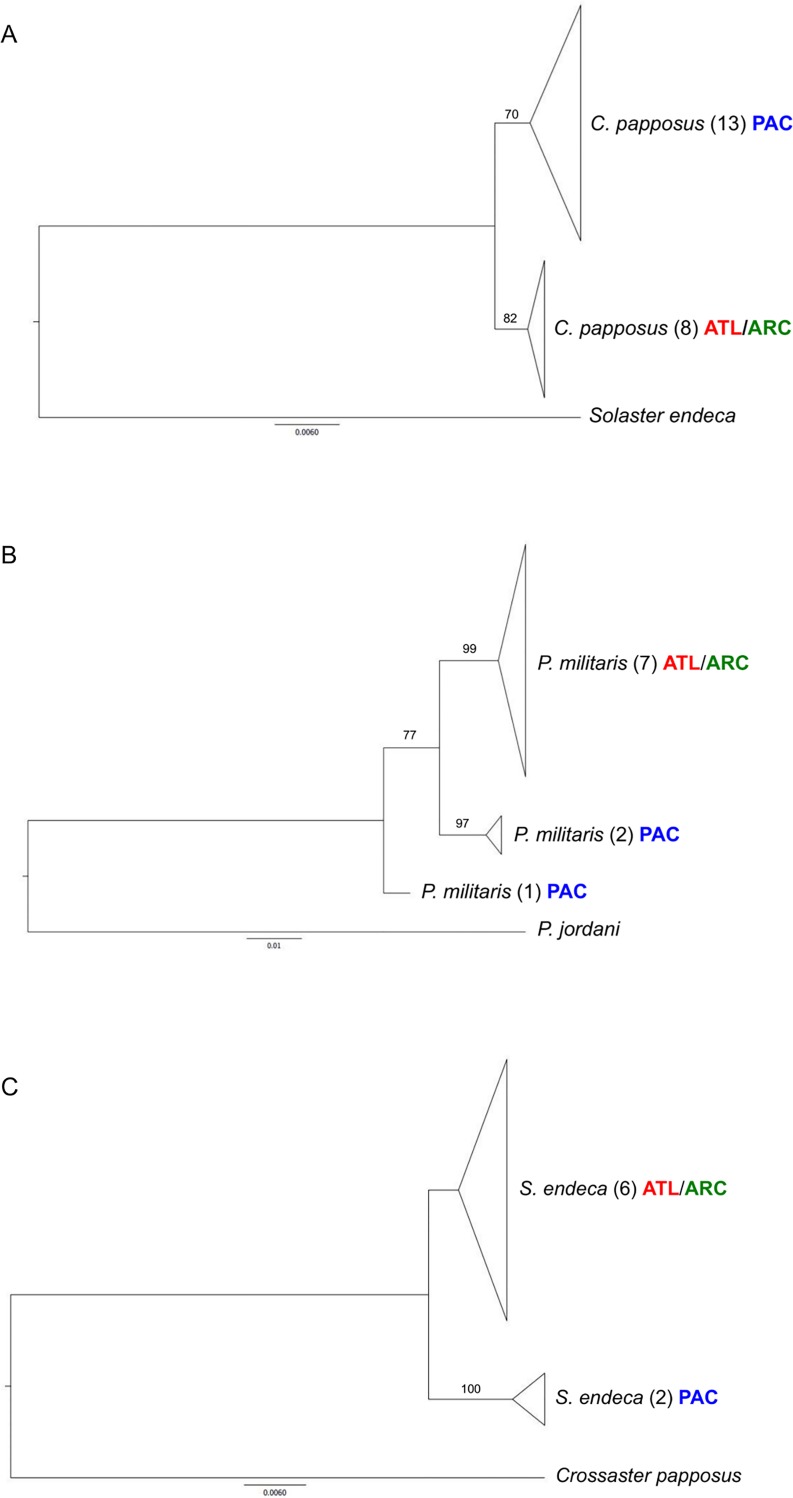
Divergences at COI for three echinoderm species. Maximum-likelihood (HKY+I) trees illustrating population structure at COI in three species of Canadian echinoderms: A) *Crossaster papposus*, B) *Pteraster militaris* and C) *Solaster endeca*. Sequence number is presented in brackets for each clade. Scale bars represent percent sequence divergence.

Twelve of 19 species collected from two or more oceans (Table 2) had low intraspecific divergence with little to no geographic structure. Two others showed clear geographic structure although their maximum divergences were below 2%, while two species had maximum divergences above 2%, but without obvious geographic partitioning. Finally, three species showed clear genetic structure and maximum divergences above 2%. NJ trees are presented for the three species with the most conspicuous geographic structure; one with a maximum divergence below 2% (*Crossaster papposus*), and two with maximum divergences above 2% (*P militaris*, *Solaster endeca*) (Fig 6). Sequences of *P*. *militaris* and *S*. *endeca* from the White Sea were included in the phylogenetic analysis and clustered with populations from the Atlantic Ocean. Divergences between lineages were relatively shallow (<3%) in the five species with geographic partitioning. Different lineages were normally allopatric, but *G*. *arcticus* was exceptional as two specimens from Nunavut clustered with Atlantic conspecifics rather than with other samples from Nunavut.

**Table 2:** Divergences at COI for echinoderm species from multiple oceans.

Group	Species	# BINs	Sample size	Maximum intraspecific divergence (%K2P)
			Pac/Arc/Atl	
**A**	*Gorgonocephalus arcticus*	2	0/11/5	2.53
	*Pteraster militaris*	3	3/0/5	2.98
	*Solaster endeca*	1	2/1/4	2.02
**B**	*Ophiopholis aculeata*	1	0/8/19	2.18
	*Ophiura sarsii*	2	3/13/0	7.58
**C**	*Crossaster papposus*	1	13/4/4	1.24
	*Leptasterias littoralis*	1	0/16/11	1.29
**D**	*Chirodota laevis*	1	0/1/5	0
	*Ctenodiscus crispatus*	1	10/15/0	0.62
	*Cucumaria frondosa*	1	0/9/8	0.98
	*Florometra serratissima*	1	8/13/0	0.80
	*Henricia oculata*	1	3/0/4	1.26
	*Henricia* sp. *AAD3482*	1	0/1/6	0.77
	*Henricia* sp. *AAB9183*	1	2/1/13	1.55
	*Ophiura robusta*	1	0/24/1	0.31
	*Psolus fabricii*	1	0/11/5	0.43
	*Psolus phantapus*	1	0/5/2	0.51
	*Strongylocentrotus droebachiensis*	1	11/0/8	0.53
	*Strongylocentrotus pallidus*	1	1/24/0	0.31

Four patterns of regional divergence in COI sequences were observed among the 19 echinoderm species analysed from two or more of Canada’s oceans. Group A: species with maximum intraspecific divergences > 2% and geographic partitioning; Group B: species with maximum intraspecific divergences > 2% and no obvious geographic partitioning; Group C: species with maximum intraspecific divergences < 2% and geographic partitioning and Group D: species with maximum intraspecific divergences < 2% and no obvious geographic partitioning. Sample sizes are provided for each locality (Pac = Pacific, Arc = Arctic, Atl = Atlantic).

## Discussion

### Barcode Recovery

The present study recovered barcode sequences from 78% of the specimens that were analyzed. The failure to recover barcodes from all specimens is likely a consequence of the fact that current primer sets are not effective for all echinoderm lineages. Although the present study expanded the primer sets [14] available, more work is needed to develop a set that recovers barcodes from all echinoderms.

### Species delineation

This study further validates the effectiveness of DNA barcoding as a tool for species discrimination in echinoderms. All 141 species examined in this study possessed diagnostic sequence variation at COI, and discrimination of species was generally straightforward because of the large difference (25-fold) between mean intra- (0.48%) and inter- (12%) specific divergence. This pattern is consistent with other studies which employed COI to examine relationships within and between echinoderm species [14,26,56–58]. Barcode data separated all currently recognized species examined in this study, and also revealed that specimens of six species were assigned to two or three BINs, suggesting possible overlooked taxa. Moreover, COI revealed evidence of phylogeographic patterns in five of the 19 species analyzed from more than one ocean. Because sample sizes for the 12 species with sequence divergences above 2% were small, further investigations are needed to clarify the taxonomic status of their component lineages. However, the present data do suggest that the current taxonomic system has not overlooked many species.

### Patterns of intraspecific divergence between allopatric populations

All nine species (*Ctenodiscus crispatus*, *Cucumaria frondosa*, *Florometra serratissima*, *O*. *sarsii*, *Psolus fabricii*, *Psolus phantapus*, *S*. *droebachiensis*, *S*. *pallidus*) which lack population structure across two or three of Canada’s oceans possess planktonic larval stages capable of long-range dispersal. However, three other species (*G*. *arcticus*, *P*. *militaris*, *S*. *endeca*) with planktonic larvae showed high intraspecific variation (max. 2–3%) at COI and clear geographic population structure. *G*. *arcticus* produces planktotrophic larvae while those of *S*. *endeca* are lecithotrophic [39]. *P*. *militaris* has a more complex reproductive strategy because females brood some young, but broadcast spawn others [59]. Because these three species produce planktonic larvae, little regional differentiation would be expected among their populations, suggesting that the deep divergences might indicate overlooked taxa. Since barcode records were obtained from both species of *Gorgonocephalus* (*G*. *arcticus*, *G*. *eucnemis*) known from Canada [60], the oversight of a known species cannot explain the deep divergence detected in *G*. *arcticu*s. The situation for the other two genera (*Pteraster*, *Solaster*) is less certain because both genera include other Canadian species that lack barcode records [61]. As a consequence, the high intraspecific divergences for *P*. *militaris* and *S*. *endeca* might reflect misidentifications. When these divergent lineages are considered as different species, mean intraspecific divergences for these two taxa drop to 0.56% and the resulting interspecific distances (2–3%), although low, are similar to those observed for COI in other closely related echinoderm species (*Echinometra*: 2–3% [30]; *Leptasterias*: 0.4–2.2%, [23]; *Patiriella* 1.1–4.3%, [29]).

The genetic divergences in *S*. *endeca* and *P*. *militaris* involve differences between Pacific and Arctic-Atlantic populations, but they are too low to reflect isolation since the 3.5mya trans-Arctic interchange based on standard rates of mtDNA evolution [24]. Instead the 2–3% divergence in these species suggests that gene flow has occurred as recently as 1–1.5 million years ago. The divergence (2.0%) between Pacific and Arctic-Atlantic populations of *S*. *endeca* is considerably higher than that (1.2%) in *C*. *papposus*, a related species with a similar mode of dispersal [39]. Moreover, because high levels of population structure have been observed over just a few tens of kilometres in marine species with low dispersal [62], the divergence in Pacific populations of *P*. *militaris* (Fig 6B) may represent cryptic speciation. Additional sampling is required to better understand divergences between populations in different ocean basins. In any case, Atlantic and Arctic populations of *C*. *papposus*, *P*. *militaris*, and *S*. *endeca* are more similar to each other than to their Pacific counterparts, a pattern noted in several other North American echinoderms [25]. Three species with maximum intraspecific divergence above 2% have population structure associated with distance, but need further investigation to clarify their taxonomic status. It is also possible that further geographic sampling particularly across the Arctic will reveal situations in which lineages occur sympatrically, allowing a test of their reproductive isolation.

### Intraspecific divergence in sympatry

Seven of the 12 species with maximum intraspecific divergences >2% have relatively narrow geographic sampling, while two (*Ophiopholis aculeata*, *O*. *sarsii*) were collected from two oceans. *L*. *clarki*, a small brooding sea cucumber collected in the NE Pacific, had a maximum intraspecific divergence of 3.07%. The single divergent sequence of *L*. *clarki* might represent *L*. *transgressor*, the other species of this genus known from this region. These closely related species are difficult to distinguish morphologically, and have occasionally been treated as synonyms [38,63]. Alternatively, this sequence could represent a new cryptic species. Although further study is required, the sequence clusters in *L*. *furcilliger* may represent cryptic species with differing depth preferences. In contrast, *O*. *aculeata* was relatively well sampled across the Arctic and Atlantic (n = 28), but no population structure was observed. Larger sample sizes with broader geographic sampling and examination of additional characters are needed to explain the higher levels of intraspecific variation observed in these species. Lastly, *L*. *hexactis* was represented by two BINs in British Columbia, a result that supports prior work which has highlighted cryptic complexes in several *Leptasterias* species using both allozyme analysis [64] and other molecular techniques [65,66].

### Patterns of divergence in a species-rich genus

Preliminary results suggest that there are over 100 species in the genus *Henricia* with particularly high levels of diversity in the NE Pacific (M. Strathmann and D. Eernisse pers. comm.). Closely related species in this genus often show subtle morphological differences and a tendency to hybridize, making identifications exceedingly difficult [40,67,68]. Although *Henricia* exhibits variable reproductive strategies ranging from brood-protection to pelagic lecithotrophy [39,69,70], challenges in species identification have made it difficult to ascertain if this variation occurs between or within species. The amphi-boreal distributions of *H*. *cf*. *oculata* and *H*. sp. *AAB9183* suggest that these species possess pelagic larvae, but do not rule out brooding because rafting by adults may also facilitate gene flow [71]. Although the present results suggest that DNA barcoding can advance understanding of species boundaries in this genus, future work should also incorporate nuclear markers to probe for evidence of hybridization.

## Conclusions

This study represents an important first step in the development of a DNA barcode library for the Canadian echinoderm fauna. However, additional sampling is needed, particularly in the deep-sea, as almost half of the fauna occur at depths greater than 200m with approximately 30% inhabiting depths greater than 1500m [37]. Continued expansion of the barcode reference library for echinoderms will greatly enhance the utility of DNA barcoding as an identification tool in support of a range of ecological, systematic and biodiversity studies.

## Supporting Information

S1 Table Specimens used in this study. List of BOLD sample IDs, process IDs, GenBank accession numbers, and institution storing for each specimen in the dataset DS-COIECH dx.doi.org/10.5883/DS-COIECH on BOLD. *sequences lacking a BIN ID
(PDF)

S2 Table Intraspecific distances. List of 113 species with two or more individuals and their corresponding mean and maximum intraspecific distances (%K2P).
(PDF)

S3 Table Locality information for each BIN. List of BINs with corresponding species ID, number of individuals, and locality information. Localities from a particular province/territory are separated by a comma, while semi-colon separates those from another province/territory. Species with multiple BIN assignments are in **BOLD**. Locality codes are as follows: Alutasivik Island (AI), Amundsen Gulf (AG), Baffin Bay (BB), Bamfield (BA), Barkley Canyon (BC), Barkley Sound (BS), Beaufort Sea (BE), Cape Scott (CS), Cape St. James (CJ), Churchill (CH), Cornwallis Island (CI), Devon Island (DI), Durban Harbour (DH), Haida Gwaii (HG), Howe Sound (HS), Hudson Bay (HB), Igloolik (IG), Kyoquot Sound (KS), Labrador Sea (LS), Lancaster Sound (LA), Nanaimo (NA), Nootka Sound (NS), Prince of Wales Strait (PW), Qikiqtarjuac (QK), Quatsino Sound (QS), Resolute (RE), Sechelt (SE), Somerset Island (SI), St. Andrews (SA), Vancouver Island (VI). *sequences lacking a BIN ID.
(PDF)
