# Supplementary material for: Exploring Canadian Echinoderm Diversity through DNA Barcodes
Source: PLoS One. 2016 Nov 21;11(11):e0166118. doi: 10.1371/journal.pone.0166118 (PMC5117606; doi:10.1371/journal.pone.0166118)
Supplement: S1 Table — List of BOLD sample IDs, process IDs, GenBank accession numbers, and institution storing for each specimen in the dataset DS-COIECH dx.doi.org/10.5883/DS-COIECH on BOLD. *sequences lacking a BIN ID (PDF) [file pone.0166118.s001.pdf]

BOLD Sample ID	BOLD Process ID	Species	GenBank Accession	Institution Storing
BAM00099	DSPEC548-08	*Amphiodia cf. urtica*	HM542070	CBG
10BIOBC-EC143	EQCS160-11	*Amphiodia cf. urtica*	KU495755	CBG
10BIOBC-EC141	EQCS158-11	*Amphiodia cf. urtica*	KU495782	CBG
BAM00097	DSPEC546-08	*Amphiodia cf. urtica*	HM542068	CBG
10BIOBC-EC140	EQCS157-11	*Amphiodia cf. urtica*	KU495895	CBG
BAM00098	DSPEC547-08	*Amphiodia cf. urtica*	HM542069	CBG
10BIOBC-EC033	EQCS092-11	*Amphiodia occidentalis*	KU495910	CBG
10BIOBC-EC056	EQCS102-11	*Amphiodia occidentalis*	KU495744	CBG
BAM00096	DSPEC545-08	*Amphioplus macraspis*	HM542065	CBG
BAM00104	DSPEC553-08	*Amphioplus macraspis*	HM542067	CBG
BAM00095	DSPEC544-08	*Amphioplus macraspis*	HM542064	CBG
BAM00023	DSPEC472-08	*Amphioplus macraspis*	HM542063	CBG
BAM00103	DSPEC552-08	*Amphioplus macraspis*	HM542066	CBG
BAM00022	DSPEC471-08	*Amphioplus macraspis*	HM542062	CBG
10BIOBC-EC092	EQCS126-11	*Amphipholis* sp. *AAD7203*	KU495846	CBG
BAM00108	DSPEC557-08	*Amphipholis* sp. *AAD7203*	HM542076	CBG
10BIOBC-EC080	EQCS114-11	*Amphipholis* sp. *AAD7203*	KU495874	CBG
BAM00107	DSPEC556-08	*Amphipholis* sp. *AAD7203*	HM542075	CBG
10BIOBC-EC099	EQCS133-11	*Amphipholis* sp. *AAD7203*	KU495778	CBG
BAM00109	DSPEC558-08	*Amphipholis* sp. *AAD7203*	HM542077	CBG
BAM00100	DSPEC549-08	*Amphipholis* sp. *AAD7203*	HM542073	CBG
10BIOBC-EC081	EQCS115-11	*Amphipholis* sp. *AAD7203*	KU495907	CBG
BAM00102	DSPEC551-08	*Amphipholis* sp. *AAD7203*	HM542074	CBG
BAM00136	DSPEC585-08	*Amphipholis* sp. *AAD7203*	HM542078	CBG
10BIOBC-EC095	EQCS129-11	*Amphipholis* sp. *AAD7203*	KU495788	CBG
BAM00068	DSPEC517-08	*Amphipholis* sp. *AAJ2302*	HM542071	CBG
BAM00105	DSPEC554-08	*Amphipholis* sp. *AAJ2302*	HM542072	CBG
10BIOBC-EC050	EQCS099-11	*Amphipholis* sp. *AAU6176*	KU495883	CBG
HUNT0043	DSPEC720-08	*Amphipholis squamata*	HM542079	CBG
HUNT0045	DSPEC722-08	*Amphipholis squamata*	HM542080	CBG
HUNT0046	DSPEC723-08	*Amphipholis squamata*	HM542081	CBG
HUNT0047	DSPEC724-08	*Amphipholis squamata*	HM542082	CBG
HUNT0048	DSPEC725-08	*Amphipholis squamata*	HM542083	CBG
CECE07-027	DSPEC097-07	*Asterias forbesi*	HM542088	CBG
HUNT0016	DSPEC693-08	*Asterias forbesi*	HM542092	CBG
CECE07-023	DSPEC093-07	*Asterias forbesi*	HM542084	CBG
CECE07-024	DSPEC094-07	*Asterias forbesi*	HM542085	CBG
HUNT0008	DSPEC685-08	*Asterias forbesi*	HM542089	CBG
CECE07-025	DSPEC095-07	*Asterias forbesi*	HM542086	CBG
HUNT0017	DSPEC694-08	*Asterias forbesi*	HM542093	CBG
CECE07-026	DSPEC096-07	*Asterias forbesi*	HM542087	CBG
HUNT0009	DSPEC686-08	*Asterias forbesi*	HM542090	CBG
HUNT0010	DSPEC687-08	*Asterias forbesi*	HM542091	CBG
HUNT0018	DSPEC695-08	*Asterias rubens*	HM542099	CBG
HUNT0020	DSPEC697-08	*Asterias rubens*	HM542101	CBG
HUNT0019	DSPEC696-08	*Asterias rubens*	HM542100	CBG
HUNT0021	DSPEC698-08	*Asterias rubens*	HM542102	CBG
CECE07-029	DSPEC099-07	*Asterias rubens*	HM542094	CBG
CECE07-030	DSPEC100-07	*Asterias rubens*	HM542095	CBG
CECE07-032	DSPEC102-07	*Asterias rubens*	HM542097	CBG
CECE07-031	DSPEC101-07	*Asterias rubens*	HM542096	CBG
HUNT0022	DSPEC699-08	*Asterias rubens*	HM542103	CBG
HUNT0023	DSPEC700-08	*Asterias rubens*	HM542104	CBG
CECE07-033	DSPEC103-07	*Asterias rubens*	HM542098	CBG
10BIOBC-EC034	EQCS093-11	*Asterina miniata*	JN259388	CBG

10BIOBC-EC011	EQCS082-11	*Asterina miniata*	KU495736	CBG
BAM00013	DSPEC462-08	*Asterina miniata*	HM542107	CBG
10BIOBC-EC012	EQCS188-11	*Asterina miniata*	KU495859	CBG
10BIOBC-EC039	EQCS221-11	*Asterina miniata*	KU495909	CBG
BAM00012	DSPEC461-08	*Asterina miniata*	HM542106	CBG
BAM00011	DSPEC460-08	*Asterina miniata*	HM542105	CBG
10BIOBC-EC129	EQCS229-11	*Asterina miniata*	KU495814	CBG
10BIOBC-EC008	EQCS187-11	*Asterina miniata*	KU495849	CBG
10BIOBC-EC038	EQCS220-11	*Asterina miniata*	KU495812	CBG
BAM00085	DSPEC534-08	*Asterina miniata*	HM542108	CBG
10BIOBC-EC040	EQCS222-11	*Asterina miniata*	KU495888	CBG
BAM00086	DSPEC535-08	*Asterina miniata*	HM542109	CBG
10BIOBC-EC035	EQCS094-11	*Asterina miniata*	KU495833	CBG
BAM00073	DSPEC522-08	*Brisaster latifrons*	HM542115	CBG
BAM00153	DSPEC602-08	*Ceramaster patagonicus*	HM542121	CBG
BAM00152	DSPEC601-08	*Ceramaster patagonicus*	HM542120	CBG
HUNT0003	DSPEC680-08	*Chiridota laevis*	HM542122	CBG
BIOUG14664-B11	ARCMI591-14	*Chiridota laevis*	KU495903	CBG
HUNT0115	DSPEC793-09	*Chiridota laevis*	HM400348	CBG
HUNT0030	DSPEC707-08	*Chiridota laevis*	HM542124	CBG
HUNT0032	DSPEC709-08	*Chiridota laevis*	HM542125	CBG
HUNT0027	DSPEC704-08	*Chiridota laevis*	HM542123	CBG
HLC-24108	EQCS023-08	*Crossaster papposus*	HM473903	CBG
CECE07-037	DSPEC107-07	*Crossaster papposus*	HM542127	CBG
HLC-24102	EQCS020-08	*Crossaster papposus*	HM473902	CBG
CECE07-039	DSPEC109-07	*Crossaster papposus*	HM542129	CBG
10BIOBC-EC086	EQCS120-11	*Crossaster papposus*	KU495771	CBG
HLC-24109	EQCS024-08	*Crossaster papposus*	HM473904	CBG
10BIOBC-EC087	EQCS121-11	*Crossaster papposus*	KU495780	CBG
INV0673_05	ECCH024-09	*Crossaster papposus*	HM473812	CBG
10BIOBC-EC085	EQCS119-11	*Crossaster papposus*	KU495829	CBG
10BIOBC-EC096	EQCS130-11	*Crossaster papposus*	KU495820	CBG
BAM00162	DSPEC611-08	*Crossaster papposus*	HM542133	CBG
HLC-30263	ECNN018-08	*Crossaster papposus*	HM543003	CBG
BAM00161	DSPEC610-08	*Crossaster papposus*	HM542132	CBG
HLC-30104	ECNN002-08	*Crossaster papposus*	HM543002	CBG
BAM00159	DSPEC608-08	*Crossaster papposus*	HM542130	CBG
CECE07-038	DSPEC108-07	*Crossaster papposus*	HM542128	CBG
CECE07-036	DSPEC106-07	*Crossaster papposus*	HM542126	CBG
BAM00163	DSPEC612-08	*Crossaster papposus*	HM542134	CBG
BAM00164	DSPEC613-08	*Crossaster papposus*	HM542135	CBG
INV0631	ECNN111-08	*Crossaster papposus*	HM473811	CBG
BAM00160	DSPEC609-08	*Crossaster papposus*	HM542131	CBG
10CHEC-011	CHEC011-11	*Ctenodiscus crispatus*	KU495847	CBG
10CHEC-021	CHEC021-11	*Ctenodiscus crispatus*	KU495804	CBG
10CHEC-023	CHEC023-11	*Ctenodiscus crispatus*	KU495891	CBG
HLC-24068	EQCS058-08	*Ctenodiscus crispatus*	HM473907	CBG
HLC-23909	EQCS032-08	*Ctenodiscus crispatus*	HM473906	CBG
HLC-23907	EQCS031-08	*Ctenodiscus crispatus*	HM473905	CBG
10CHEC-076	CHEC066-12	*Ctenodiscus crispatus*	KU495809	CBG
09CHON-009	CHONE009-10	*Ctenodiscus crispatus*	HM405877	CBG
10CHEC-018	CHEC018-11	*Ctenodiscus crispatus*	KU495796	CBG
HLC-24198	EQCS068-08	*Ctenodiscus crispatus*	HM473908	CBG
10CHEC-024	CHEC024-11	*Ctenodiscus crispatus*	KU495884	CBG
09CHON-010	CHONE010-10	*Ctenodiscus crispatus*	HM405878	CBG
10CHEC-012	CHEC012-11	*Ctenodiscus crispatus*	KU495898	CBG
10CHEC-072	CHEC062-12	*Ctenodiscus crispatus*	KU495877	CBG
10CHEC-010	CHEC010-11	*Ctenodiscus crispatus*	KU495857	CBG

10CHEC-077	CHEC067-12	*Ctenodiscus crispatus*	KU495740	CBG
10CHEC-022	CHEC022-11	*Ctenodiscus crispatus*	KU495749	CBG
10CHEC-025	CHEC025-11	*Ctenodiscus crispatus*	KU495836	CBG
HLC-24199	EQCS069-08	*Ctenodiscus crispatus*	HM473909	CBG
09CHON-008	CHONE008-10	*Ctenodiscus crispatus*	HM405876	CBG
BAM00024	DSPEC473-08	*Cucumaria cf. lubrica*	HM542141	CBG
10BIOBC-00622	EQCS164-11	*Cucumaria cf. lubrica*	KU495917	CBG
BAM00025	DSPEC474-08	*Cucumaria cf. lubrica*	HM542142	CBG
CECE07-040	DSPEC110-07	*Cucumaria frondosa*	HM542143	CBG
BIOUG14666-C04	ARCMI406-14	*Cucumaria frondosa*	KU495835	CBG
HUNT0035	DSPEC712-08	*Cucumaria frondosa*	HM542149	CBG
CECE07-042	DSPEC112-07	*Cucumaria frondosa*	HM542145	CBG
HLC-30467	ECNN077-08	*Cucumaria frondosa*	HM543012	CBG
HLC-L19402	ECNN075-08	*Cucumaria frondosa*	HM543011	CBG
HLC-L13401	ECNN053-08	*Cucumaria frondosa*	HM543008	CBG
CECE07-043	DSPEC113-07	*Cucumaria frondosa*	HM542146	CBG
HLC-30109	ECNN003-08	*Cucumaria frondosa*	HM543004	CBG
HLC-30301	ECNN017-08	*Cucumaria frondosa*	HM543005	CBG
CECE07-044	DSPEC114-07	*Cucumaria frondosa*	HM542147	CBG
HLC-L13402	ECNN054-08	*Cucumaria frondosa*	HM543009	CBG
CECE07-045	DSPEC115-07	*Cucumaria frondosa*	HM542148	CBG
HLC-L19401	ECNN074-08	*Cucumaria frondosa*	HM543010	CBG
HLC-30302	ECNN049-08	*Cucumaria frondosa*	HM543007	CBG
HLC-30166	ECNN048-08	*Cucumaria frondosa*	HM543006	CBG
CECE07-041	DSPEC111-07	*Cucumaria frondosa*	HM542144	CBG
HUNT0036	DSPEC713-08	*Cucumaria frondosa*	HM542150	CBG
10BIOBC-EC036	EQCS095-11	*Cucumaria miniata*	KU495887	CBG
BAM00037	DSPEC486-08	*Cucumaria miniata*	HM542157	CBG
BIOUG00903-A03	EQCS230-12	*Cucumaria miniata*	KU495806	CBG
10BIOBC-EC009	EQCS080-11	*Cucumaria miniata*	KU495837	CBG
BAM00028	DSPEC477-08	*Cucumaria miniata*	HM542153	CBG
BAM00026	DSPEC475-08	*Cucumaria miniata*	HM542151	CBG
BAM00036	DSPEC485-08	*Cucumaria miniata*	HM542156	CBG
BAM00029	DSPEC478-08	*Cucumaria miniata*	HM542154	CBG
10BIOBC-EC001	EQCS185-11	*Cucumaria miniata*	KU495805	CBG
BAM00027	DSPEC476-08	*Cucumaria miniata*	HM542152	CBG
BAM00035	DSPEC484-08	*Cucumaria miniata*	HM542155	CBG
BAM00180	DSPEC629-08	*Cucumaria pallida*	HM542158	CBG
BAM00110*	DSPEC559-08	*Cucumaria pallida*	HM542160	CBG
BAM00079	DSPEC528-08	*Cucumaria pallida*	HM542159	CBG
10BIOBC-EC089	EQCS123-11	*Cucumaria pallida*	KU495886	CBG
10BIOBC-EC090	EQCS124-11	*Cucumaria piperata*	KU495785	CBG
10BIOBC-EC116	EQCS144-11	*Cucumaria pseudocurata*	KU495889	CBG
10BIOBC-EC112	EQCS140-11	*Cucumaria pseudocurata*	KU495845	CBG
10BIOBC-EC118	EQCS146-11	*Cucumaria pseudocurata*	KU495825	CBG
10BIOBC-EC115	EQCS143-11	*Cucumaria pseudocurata*	KU495739	CBG
10BIOBC-EC117	EQCS145-11	*Cucumaria pseudocurata*	KU495826	CBG
10BIOBC-EC111	EQCS139-11	*Cucumaria pseudocurata*	KU495890	CBG
10BIOBC-EC114	EQCS142-11	*Cucumaria pseudocurata*	KU495791	CBG
10BIOBC-EC113	EQCS141-11	*Cucumaria pseudocurata*	KU495800	CBG
BAM00221	DSPEC670-08	*Dendraster excentricus*	HM542162	CBG
BAM00218	DSPEC667-08	*Dendraster excentricus*	GU670169	CBG
BAM00219	DSPEC668-08	*Dendraster excentricus*	GU670168	CBG
BAM00222	DSPEC671-08	*Dendraster excentricus*	HM542163	CBG
BAM00220	DSPEC669-08	*Dendraster excentricus*	HM542161	CBG
10BIOBC-EC032	EQCS190-11	*Dermasterias imbricata*	KU495818	CBG
BAM00080	DSPEC529-08	*Dermasterias imbricata*	HM542166	CBG
BAM00084	DSPEC533-08	*Dermasterias imbricata*	HM542170	CBG

BAM00202	DSPEC651-08	*Dermasterias imbricata*	HM542165	CBG
BAM00082	DSPEC531-08	*Dermasterias imbricata*	HM542168	CBG
BAM00083	DSPEC532-08	*Dermasterias imbricata*	HM542169	CBG
10BIOBC-EC017	EQCS212-11	*Dermasterias imbricata*	KU495822	CBG
10BIOBC-EC136	EQCS207-11	*Dermasterias imbricata*	KU495842	CBG
10BIOBC-00621	EQCS163-11	*Dermasterias imbricata*	KU495870	CBG
BAM00201	DSPEC650-08	*Dermasterias imbricata*	HM542164	CBG
10BIOBC-EC060	EQCS180-11	*Dermasterias imbricata*	KU495848	CBG
10BIOBC-EC134	EQCS153-11	*Dermasterias imbricata*	KU495840	CBG
BAM00081	DSPEC530-08	*Dermasterias imbricata*	HM542167	CBG
CECE07-006	DSPEC076-07	*Echinarachnius parma*	HM542171	CBG
CECE07-007	DSPEC077-07	*Echinarachnius parma*	HM542172	CBG
HUNT0038	DSPEC715-08	*Echinarachnius parma*	HM542175	CBG
CECE07-010	DSPEC080-07	*Echinarachnius parma*	HM542174	CBG
CECE07-009	DSPEC079-07	*Echinarachnius parma*	HM542173	CBG
07PROBE-ECH026	DSPEC070-07	*Ekmania barthii*	HM473813	CBG
BAM00129	DSPEC578-08	*Eupentacta quinquesemita*	HM542179	CBG
BAM00127	DSPEC576-08	*Eupentacta quinquesemita*	HM542177	CBG
BAM00033	DSPEC482-08	*Eupentacta quinquesemita*	HM542176	CBG
BAM00128	DSPEC577-08	*Eupentacta quinquesemita*	HM542178	CBG
BAM00088	DSPEC537-08	*Evasterias troscheli*	HM542185	CBG
10BIOBC-EC020	EQCS215-11	*Evasterias troscheli*	KU495912	CBG
10BIOBC-EC022	EQCS086-11	*Evasterias troscheli*	KU495792	CBG
10BIOBC-EC135	EQCS154-11	*Evasterias troscheli*	KU495865	CBG
10BIOBC-EC137	EQCS208-11	*Evasterias troscheli*	KU495905	CBG
BAM00041	DSPEC490-08	*Evasterias troscheli*	HM542182	CBG
10BIOBC-EC013	EQCS211-11	*Evasterias troscheli*	KU495750	CBG
BAM00053	DSPEC502-08	*Evasterias troscheli*	HM542183	CBG
BAM00087	DSPEC536-08	*Evasterias troscheli*	HM542184	CBG
BAM00092	DSPEC541-08	*Evasterias troscheli*	HM542187	CBG
BAM00021	DSPEC470-08	*Evasterias troscheli*	HM542181	CBG
BAM00091	DSPEC540-08	*Evasterias troscheli*	HM542186	CBG
10BIOBC-EC021	EQCS085-11	*Evasterias troscheli*	KU495738	CBG
BAM00177	DSPEC626-08	*Florometra serratissima*	HM542190	CBG
HLC-30131	ECNN030-08	*Florometra serratissima*	HM543014	CBG
10BIOBC-EC103	EQCS184-11	*Florometra serratissima*	KU495893	CBG
HLC-30321	ECNN041-08	*Florometra serratissima*	HM543016	CBG
BAM00175	DSPEC624-08	*Florometra serratissima*	HM542188	CBG
09CHON-002	CHONE002-10	*Florometra serratissima*	HM405870	CBG
10CHEC-004	CHEC004-11	*Florometra serratissima*	KU495747	CBG
10CHEC-034	CHEC034-11	*Florometra serratissima*	KU495752	CBG
BAM00176	DSPEC625-08	*Florometra serratissima*	HM542189	CBG
10CHEC-003	CHEC003-11	*Florometra serratissima*	KU495834	CBG
10CHEC-001	CHEC001-11	*Florometra serratissima*	KU495779	CBG
10BIOBC-EC101	EQCS199-11	*Florometra serratissima*	KU495799	CBG
BAM00178	DSPEC627-08	*Florometra serratissima*	HM542191	CBG
10CHEC-002	CHEC002-11	*Florometra serratissima*	KU495784	CBG
HLC-30245	ECNN019-08	*Florometra serratissima*	HM543013	CBG
10BIOBC-EC102	EQCS225-11	*Florometra serratissima*	KU495852	CBG
BAM00179	DSPEC628-08	*Florometra serratissima*	HM542192	CBG
10CHEC-005	CHEC005-11	*Florometra serratissima*	KU495855	CBG
HLC-30140	ECNN032-08	*Florometra serratissima*	HM543015	CBG
10CHEC-033	CHEC033-11	*Florometra serratissima*	KU495885	CBG
10CHEC-035	CHEC035-11	*Florometra serratissima*	KU495860	CBG
CECE07-003	DSPEC073-07	*Gorgonocephalus arcticus*	HM542195	CBG
10CHEC-029	CHEC029-11	*Gorgonocephalus arcticus*	KU495828	CBG
HLC-30309	ECNN040-08	*Gorgonocephalus arcticus*	HM543017	CBG
10CHEC-031	CHEC031-11	*Gorgonocephalus arcticus*	KU495810	CBG

CECE07-002	DSPEC072-07	*Gorgonocephalus arcticus*	HM542194	CBG
10CHEC-028	CHEC028-11	*Gorgonocephalus arcticus*	KU495867	CBG
10CHEC-032	CHEC032-11	*Gorgonocephalus arcticus*	KU495816	CBG
HUNT0041	DSPEC718-08	*Gorgonocephalus arcticus*	HM542197	CBG
HLC-L15002	ECNN086-08	*Gorgonocephalus arcticus*	HM543019	CBG
HUNT0040	DSPEC717-08	*Gorgonocephalus arcticus*	HM542196	CBG
10CHEC-007	CHEC007-11	*Gorgonocephalus arcticus*	KU495765	CBG
10CHEC-006	CHEC006-11	*Gorgonocephalus arcticus*	KU495773	CBG
CECE07-001	DSPEC071-07	*Gorgonocephalus arcticus*	HM542193	CBG
10CHEC-027	CHEC027-11	*Gorgonocephalus arcticus*	KU495901	CBG
10CHEC-030	CHEC030-11	*Gorgonocephalus arcticus*	KU495753	CBG
HLC-30307	ECNN050-08	*Gorgonocephalus arcticus*	HM543018	CBG
L#QCS-018	EQCS076-08	*Gorgonocephalus eucnemis*	GU670194	CBG
HLC-24077	EQCS013-08	*Gorgonocephalus eucnemis*	HM473910	CBG
HLC-24191	EQCS063-08	*Gorgonocephalus eucnemis*	HM473911	CBG
BAM00181	DSPEC630-08	*Gorgonocephalus eucnemis*	HM542198	CBG
HLC-23917	EQCS037-08	*Henricia leviuscula spiculifera*	GU670191	CBG
HLC-24126	EQCS028-08	*Henricia oculata*	HM473912	CBG
HUNT0087	DSPEC765-09	*Henricia oculata*	HM400336	CBG
HLC-23960	EQCS041-08	*Henricia oculata*	HM473913	CBG
CECE07-016	DSPEC086-07	*Henricia oculata*	GU670164	CBG
HUNT0062	DSPEC739-08	*Henricia oculata*	GU670162	CBG
HLC-23962	EQCS043-08	*Henricia oculata*	HM473914	CBG
HUNT0088	DSPEC766-09	*Henricia oculata*	HM400337	CBG
HUNT0079*	DSPEC757-09	*Henricia sanguinolenta*	HM542200	CBG
HUNT0061	DSPEC738-08	*Henricia sanguinolenta*	HM542199	CBG
HUNT0084	DSPEC762-09	*Henricia* sp. *AAB3569*	HM400333	CBG
HUNT0082	DSPEC760-09	*Henricia* sp. *AAB3569*	HM400331	CBG
HUNT0095	DSPEC773-09	*Henricia* sp. *AAB3569*	HM400341	CBG
HUNT0068	DSPEC745-08	*Henricia* sp. *AAB3569*	HM542210	CBG
HUNT0137	DSPEC815-09	*Henricia* sp. *AAB3569*	HM542211	CBG
HUNT0152	DSPEC830-09	*Henricia* sp. *AAB3569*	HM400368	CBG
HUNT0145	DSPEC823-09	*Henricia* sp. *AAB3569*	HM400364	CBG
HUNT0091	DSPEC769-09	*Henricia* sp. *AAB3569*	HM400339	CBG
HUNT0136	DSPEC814-09	*Henricia* sp. *AAB3569*	HM400357	CBG
CECE07-017	DSPEC087-07	*Henricia* sp. *AAB3569*	HM542208	CBG
HUNT0128	DSPEC806-09	*Henricia* sp. *AAB3569*	HM400352	CBG
HUNT0130	DSPEC808-09	*Henricia* sp. *AAB3569*	HM400354	CBG
HUNT0129	DSPEC807-09	*Henricia* sp. *AAB3569*	HM400353	CBG
HUNT0111	DSPEC789-09	*Henricia* sp. *AAB3569*	HM400347	CBG
HUNT0140	DSPEC818-09	*Henricia* sp. *AAB3569*	HM400360	CBG
CECE07-018	DSPEC088-07	*Henricia* sp. *AAB3569*	HM542209	CBG
HUNT0134	DSPEC812-09	*Henricia* sp. *AAB3569*	HM400355	CBG
HUNT0135	DSPEC813-09	*Henricia* sp. *AAB3569*	HM400356	CBG
HUNT0141	DSPEC819-09	*Henricia* sp. *AAB3569*	HM400361	CBG
HUNT0139	DSPEC817-09	*Henricia* sp. *AAB3569*	HM400359	CBG
HUNT0147	DSPEC825-09	*Henricia* sp. *AAB3569*	HM400365	CBG
HUNT0155	DSPEC833-09	*Henricia* sp. *AAB3569*	HM542212	CBG
HUNT0083	DSPEC761-09	*Henricia* sp. *AAB3569*	HM400332	CBG
HUNT0148	DSPEC826-09	*Henricia* sp. *AAB3569*	GU670167	CBG
09CHON-007	CHONE007-10	*Henricia* sp. *AAB9183*	HM405875	CBG
HUNT0110	DSPEC788-09	*Henricia* sp. *AAB9183*	HM400346	CBG
HUNT0098	DSPEC776-09	*Henricia* sp. *AAB9183*	HM400342	CBG
HLC-24125	EQCS027-08	*Henricia* sp. *AAB9183*	HM473916	CBG
HLC-24069	EQCS010-08	*Henricia* sp. *AAB9183*	HM473915	CBG
HUNT0089	DSPEC767-09	*Henricia* sp. *AAB9183*	HM400338	CBG
HUNT0117	DSPEC795-09	*Henricia* sp. *AAB9183*	HM400349	CBG
HUNT0105	DSPEC783-09	*Henricia* sp. *AAB9183*	HM400345	CBG

HUNT0085	DSPEC763-09	*Henricia* sp. *AAB9183*	HM400334	CBG
HUNT0138	DSPEC816-09	*Henricia* sp. *AAB9183*	HM400358	CBG
HUNT0104	DSPEC782-09	*Henricia* sp. *AAB9183*	HM400344	CBG
HUNT0070	DSPEC747-08	*Henricia* sp. *AAB9183*	GU670163	CBG
HUNT0100	DSPEC778-09	*Henricia* sp. *AAB9183*	HM400343	CBG
HUNT0156	DSPEC834-09	*Henricia* sp. *AAB9183*	HM400369	CBG
HUNT0149	DSPEC827-09	*Henricia* sp. *AAB9183*	HM400366	CBG
HUNT0092	DSPEC770-09	*Henricia* sp. *AAB9183*	HM400340	CBG
HUNT0123	DSPEC801-09	*Henricia* sp. *AAD3482*	HM400350	CBG
HLC-30244	ECNN022-08	*Henricia* sp. *AAD3482*	HM543020	CBG
HUNT0076	DSPEC753-08	*Henricia* sp. *AAD3482*	HM542206	CBG
HUNT0086	DSPEC764-09	*Henricia* sp. *AAD3482*	HM400335	CBG
HUNT0150	DSPEC828-09	*Henricia* sp. *AAD3482*	HM400367	CBG
CECE07-052	DSPEC122-07	*Henricia* sp. *AAD3482*	HM542205	CBG
HUNT0146	DSPEC824-09	*Henricia* sp. *AAD3482*	HM542207	CBG
BAM00133	DSPEC582-08	*Henricia* sp. *AAF2468*	HM542218	CBG
10BIOBC-EC082	EQCS116-11	*Henricia* sp. *AAF2468*	JF891307	CBG
10BIOBC-EC084	EQCS118-11	*Henricia* sp. *AAF2468*	JF891309	CBG
10BIOBC-EC066	EQCS106-11	*Henricia* sp. *AAF2468*	JF891306	CBG
BAM00045	DSPEC494-08	*Henricia* sp. *AAF2468*	HM542216	CBG
BAM00048	DSPEC497-08	*Henricia* sp. *AAF2468*	HM542217	CBG
10BIOBC-EC104	EQCS135-11	*Henricia* sp. *AAF2496*	JF891311	CBG
BAM00167	DSPEC616-08	*Henricia* sp. *AAF2496*	HM542203	CBG
BAM00168	DSPEC617-08	*Henricia* sp. *AAF2496*	HM542204	CBG
10BIOBC-EC139	EQCS156-11	*Henricia* sp. *AAI1811*	KU495880	CBG
10BIOBC-EC132	EQCS205-11	*Henricia* sp. *AAI1811*	KU495911	CBG
10BIOBC-EC122	EQCS149-11	*Henricia* sp. *AAI1811*	JF891312	CBG
10BIOBC-EC014	EQCS083-11	*Henricia* sp. *AAI1811*	JF891304	CBG
10BIOBC-EC126	EQCS202-11	*Henricia* sp. *AAI1811*	KU495876	CBG
10BIOBC-EC138	EQCS155-11	*Henricia* sp. *AAI1811*	KU495839	CBG
BAM00126	DSPEC575-08	*Henricia* sp. *AAI1811*	HM542214	CBG
10BIOBC-EC015	EQCS084-11	*Henricia* sp. *AAI1811*	JF891305	CBG
10BIOBC-EC016	EQCS189-11	*Henricia* sp. *AAI1812*	JF891316	CBG
10BIOBC-EC057	EQCS176-11	*Henricia* sp. *AAI1812*	JF891314	CBG
10BIOBC-EC044	EQCS173-11	*Henricia* sp. *AAI1812*	JF891313	CBG
10BIOBC-EC004	EQCS186-11	*Henricia* sp. *AAI1815*	JF891315	CBG
10BIOBC-EC083	EQCS117-11	*Henricia* sp. *AAI1816*	JF891308	CBG
BIOUG00903-A04	EQCS231-12	*Henricia* sp. *AAI1816*	KU495866	CBG
10BIOBC-EC098	EQCS132-11	*Henricia* sp. *AAI1816*	JF891310	CBG
BAM00043	DSPEC492-08	*Henricia* sp. *AAI1817*	GU670165	CBG
BAM00044	DSPEC493-08	*Henricia* sp. *AAI1817*	HM542215	CBG
BAM00169	DSPEC618-08	*Henricia* sp. *AAI6792*	HM542201	CBG
BAM00166	DSPEC615-08	*Henricia* sp. *AAI6792*	HM542202	CBG
10CHEC-020	CHEC020-11	*Hymenaster pellucidus*	KU495741	CBG
10CHEC-019	CHEC019-11	*Hymenaster pellucidus*	KU495861	CBG
BAM00112	DSPEC561-08	*Leptasterias hexactis*	HM542220	CBG
BAM00116	DSPEC565-08	*Leptasterias hexactis*	HM542224	CBG
BAM00118	DSPEC567-08	*Leptasterias hexactis*	HM542226	CBG
10BIOBC-EC051	EQCS100-11	*Leptasterias hexactis*	KU495894	CBG
BAM00123	DSPEC572-08	*Leptasterias hexactis*	HM542231	CBG
10BIOBC-EC125	EQCS152-11	*Leptasterias hexactis*	KU495770	CBG
10BIOBC-EC030	EQCS090-11	*Leptasterias hexactis*	KU495735	CBG
10BIOBC-EC123	EQCS150-11	*Leptasterias hexactis*	KU495830	CBG
BAM00119	DSPEC568-08	*Leptasterias hexactis*	HM542227	CBG
10BIOBC-EC052	EQCS195-11	*Leptasterias hexactis*	KU495734	CBG
10BIOBC-EC124	EQCS151-11	*Leptasterias hexactis*	KU495899	CBG
10BIOBC-EC028	EQCS219-11	*Leptasterias hexactis*	KU495914	CBG
BAM00111	DSPEC560-08	*Leptasterias hexactis*	HM542219	CBG

10BIOBC-EC055	EQCS101-11	*Leptasterias hexactis*	KU495824	CBG
BAM00117	DSPEC566-08	*Leptasterias hexactis*	HM542225	CBG
10BIOBC-EC121	EQCS148-11	*Leptasterias hexactis*	KU495742	CBG
10BIOBC-EC029	EQCS089-11	*Leptasterias hexactis*	KU495854	CBG
BAM00115	DSPEC564-08	*Leptasterias hexactis*	HM542223	CBG
10BIOBC-EC037	EQCS096-11	*Leptasterias hexactis*	KU495827	CBG
10BIOBC-EC053	EQCS174-11	*Leptasterias hexactis*	KU495823	CBG
BAM00113	DSPEC562-08	*Leptasterias hexactis*	HM542221	CBG
BAM00124	DSPEC573-08	*Leptasterias hexactis*	HM542232	CBG
BAM00121	DSPEC570-08	*Leptasterias hexactis*	HM542229	CBG
BAM00122	DSPEC571-08	*Leptasterias hexactis*	HM542230	CBG
BAM00120	DSPEC569-08	*Leptasterias hexactis*	HM542228	CBG
BAM00125	DSPEC574-08	*Leptasterias hexactis*	HM542233	CBG
10BIOBC-EC031	EQCS091-11	*Leptasterias hexactis*	KU495902	CBG
BAM00114	DSPEC563-08	*Leptasterias hexactis*	HM542222	CBG
HUNT0005	DSPEC682-08	*Leptasterias littoralis*	HM542235	CBG
HUNT0013	DSPEC690-08	*Leptasterias littoralis*	HM542240	CBG
08PROBE-2041	ECCH015-09	*Leptasterias littoralis*	HM473819	CBG
HUNT0024	DSPEC701-08	*Leptasterias littoralis*	HM542243	CBG
07PROBE-ECH009	DSPEC053-07	*Leptasterias littoralis*	HM473817	CBG
HLC-30300	ECNN016-08	*Leptasterias littoralis*	HM543022	CBG
HLC-30055	ECNN005-08	*Leptasterias littoralis*	HM543021	CBG
HUNT0012	DSPEC689-08	*Leptasterias littoralis*	HM542239	CBG
INV0671_01	ECCH027-09	*Leptasterias littoralis*	HM473820	CBG
HLC-30287	ECNN038-08	*Leptasterias littoralis*	HM543025	CBG
HUNT0015	DSPEC692-08	*Leptasterias littoralis*	HM542242	CBG
07PROBE-ECH008	DSPEC052-07	*Leptasterias littoralis*	HM473816	CBG
HUNT0014	DSPEC691-08	*Leptasterias littoralis*	HM542241	CBG
HUNT0127	DSPEC805-09	*Leptasterias littoralis*	HM400351	CBG
HLC-30447	ECNN090-08	*Leptasterias littoralis*	HM543027	CBG
HLC-30090	ECNN023-08	*Leptasterias littoralis*	HM543023	CBG
HUNT0006	DSPEC683-08	*Leptasterias littoralis*	HM542236	CBG
INV0654_01	ECNN120-08	*Leptasterias littoralis*	HM473814	CBG
07PROBE-ECH007	DSPEC051-07	*Leptasterias littoralis*	HM473815	CBG
HUNT0011	DSPEC688-08	*Leptasterias littoralis*	HM542238	CBG
INV0654_02	ECNN121-08	*Leptasterias littoralis*	GU670174	CBG
HUNT0007	DSPEC684-08	*Leptasterias littoralis*	HM542237	CBG
HUNT0004	DSPEC681-08	*Leptasterias littoralis*	HM542234	CBG
HLC-30785	ECNN160-09	*Leptasterias littoralis*	HM543028	CBG
HLC-30242	ECNN028-08	*Leptasterias littoralis*	HM543024	CBG
07PROBE-ECH017	DSPEC061-07	*Leptasterias littoralis*	HM473818	CBG
HLC-L20701	ECNN088-08	*Leptasterias littoralis*	HM543026	CBG
07PROBE-ECH003	DSPEC047-07	*Leptasterias polaris*	HM473832	CBG
07PROBE-ECH013	DSPEC057-07	*Leptasterias polaris*	HM473836	CBG
07PROBE-ECH004	DSPEC048-07	*Leptasterias polaris*	HM473833	CBG
08PROBE211_02	ECCH006-09	*Leptasterias polaris*	HM473841	CBG
INV0666	ECNN134-08	*Leptasterias polaris*	HM473823	CBG
INV0662_01	ECNN122-08	*Leptasterias polaris*	GU670177	CBG
07PROBE-ECH015	DSPEC059-07	*Leptasterias polaris*	HM473838	CBG
07PROBE-ECH005	DSPEC049-07	*Leptasterias polaris*	HM473834	CBG
07PROBE-ECH001	DSPEC045-07	*Leptasterias polaris*	HM473830	CBG
INV0665_01	ECNN130-08	*Leptasterias polaris*	HM473821	CBG
INV0668	ECNN133-08	*Leptasterias polaris*	HM473824	CBG
INV0672_06	ECNN141-08	*Leptasterias polaris*	HM473827	CBG
INV0662_02	ECNN123-08	*Leptasterias polaris*	GU670176	CBG
08PROBE211_01	ECCH005-09	*Leptasterias polaris*	HM473840	CBG
07PROBE-ECH014	DSPEC058-07	*Leptasterias polaris*	HM473837	CBG
07PROBE-ECH006	DSPEC050-07	*Leptasterias polaris*	HM473835	CBG

INV0672_04	ECNN139-08	*Leptasterias polaris*	HM473825	CBG
HLC-30046	ECNN010-08	*Leptasterias polaris*	HM543030	CBG
INV0671_02	ECCH028-09	*Leptasterias polaris*	HM473845	CBG
INV0673_06	ECCH025-09	*Leptasterias polaris*	HM473843	CBG
08PROBE-1897	ECCH013-09	*Leptasterias polaris*	HM473842	CBG
INV0671_05	ECCH031-09	*Leptasterias polaris*	HM473848	CBG
INV0665_02	ECNN131-08	*Leptasterias polaris*	HM473822	CBG
INV0672_07	ECNN142-08	*Leptasterias polaris*	HM473828	CBG
INV0672_05	ECNN140-08	*Leptasterias polaris*	HM473826	CBG
INV0671_06	ECCH032-09	*Leptasterias polaris*	HM473849	CBG
INV0671_04	ECCH030-09	*Leptasterias polaris*	HM473847	CBG
INV0673_07	ECCH026-09	*Leptasterias polaris*	HM473844	CBG
07PROBE-ECH016	DSPEC060-07	*Leptasterias polaris*	HM473839	CBG
07PROBE-ECH002	DSPEC046-07	*Leptasterias polaris*	HM473831	CBG
INV0673_04	ECNN146-08	*Leptasterias polaris*	HM473829	CBG
INV0671_03	ECCH029-09	*Leptasterias polaris*	HM473846	CBG
HLC-30105	ECNN001-08	*Leptasterias polaris*	HM543029	CBG
BAM00003	DSPEC452-08	*Leptosynapta clarki*	HM542246	CBG
BAM00008	DSPEC457-08	*Leptosynapta clarki*	HM542251	CBG
BAM00006	DSPEC455-08	*Leptosynapta clarki*	HM542249	CBG
BAM00010	DSPEC459-08	*Leptosynapta clarki*	HM542253	CBG
BAM00002	DSPEC451-08	*Leptosynapta clarki*	HM542245	CBG
BAM00007	DSPEC456-08	*Leptosynapta clarki*	HM542250	CBG
BAM00004	DSPEC453-08	*Leptosynapta clarki*	HM542247	CBG
BAM00009	DSPEC458-08	*Leptosynapta clarki*	HM542252	CBG
BAM00005	DSPEC454-08	*Leptosynapta clarki*	HM542248	CBG
BAM00001	DSPEC450-08	*Leptosynapta clarki*	HM542244	CBG
HLC-23979	EQCS046-08	*Leptychaster pacificus*	HM473918	CBG
HLC-24066	EQCS009-08	*Leptychaster pacificus*	HM473917	CBG
HLC-23986	EQCS048-08	*Lophaster furcilliger*	HM473920	CBG
HLC-23914	EQCS035-08	*Lophaster furcilliger*	HM473919	CBG
HLC-24123	EQCS025-08	*Luidia foliolata*	HM473921	CBG
HLC-24124	EQCS026-08	*Luidia foliolata*	HM473922	CBG
HLC-24194	EQCS065-08	*Luidia foliolata*	HM473926	CBG
HLC-24195	EQCS066-08	*Luidia foliolata*	HM473927	CBG
HLC-23993	EQCS055-08	*Luidia foliolata*	HM473925	CBG
HLC-23991	EQCS053-08	*Luidia foliolata*	HM473923	CBG
HLC-23992	EQCS054-08	*Luidia foliolata*	HM473924	CBG
HLC-23915	EQCS036-08	*Mediaster aequalis*	HM473928	CBG
HLC-24084	EQCS060-08	*Mediaster aequalis*	GU670193	CBG
BAM00148	DSPEC597-08	*Mediaster aequalis*	HM542268	CBG
BAM00150	DSPEC599-08	*Mediaster aequalis*	HM542270	CBG
BAM00151	DSPEC600-08	*Mediaster aequalis*	HM542271	CBG
HLC-24209	EQCS072-08	*Mediaster aequalis*	HM473930	CBG
BAM00020	DSPEC469-08	*Mediaster aequalis*	HM542266	CBG
10BIOBC-EC074	EQCS224-11	*Mediaster aequalis*	KU495858	CBG
10BIOBC-EC094	EQCS128-11	*Mediaster aequalis*	KU495768	CBG
BAM00149	DSPEC598-08	*Mediaster aequalis*	HM542269	CBG
10BIOBC-EC073	EQCS198-11	*Mediaster aequalis*	KU495748	CBG
10BIOBC-EC128	EQCS228-11	*Mediaster aequalis*	KU495811	CBG
BAM00018	DSPEC467-08	*Mediaster aequalis*	HM542264	CBG
BAM00147	DSPEC596-08	*Mediaster aequalis*	HM542267	CBG
BAM00019	DSPEC468-08	*Mediaster aequalis*	HM542265	CBG
HLC-24208	EQCS071-08	*Mediaster aequalis*	HM473929	CBG
10BIOBC-EC079	EQCS113-11	*Mediaster aequalis*	KU495853	CBG
10BIOBC-EC100	EQCS134-11	*Mediaster aequalis*	KU495743	CBG
10BIOBC-EC078	EQCS112-11	*Mesocentrotus franciscanus*	KU495881	CBG
10BIOBC-EC068	EQCS108-11	*Mesocentrotus franciscanus*	KU495878	CBG

BAM00059	DSPEC508-08	*Mesocentrotus franciscanus*	HM542403	CBG
BAM00213	DSPEC662-08	*Mesocentrotus franciscanus*	HM542396	CBG
BAM00214	DSPEC663-08	*Mesocentrotus franciscanus*	HM542397	CBG
BAM00216	DSPEC665-08	*Mesocentrotus franciscanus*	HM542399	CBG
BAM00060	DSPEC509-08	*Mesocentrotus franciscanus*	HM542404	CBG
10BIOBC-EC072	EQCS223-11	*Mesocentrotus franciscanus*	KU495841	CBG
10BIOBC-EC067	EQCS107-11	*Mesocentrotus franciscanus*	KU495896	CBG
BAM00058	DSPEC507-08	*Mesocentrotus franciscanus*	HM542402	CBG
BAM00061	DSPEC510-08	*Mesocentrotus franciscanus*	HM542405	CBG
BAM00215	DSPEC664-08	*Mesocentrotus franciscanus*	HM542398	CBG
BAM00034	DSPEC483-08	*Mesocentrotus franciscanus*	HM542401	CBG
BAM00217	DSPEC666-08	*Mesocentrotus franciscanus*	HM542400	CBG
BAM00069	DSPEC518-08	*Molpadia intermedia*	HM542277	CBG
HLC-24082	EQCS059-08	*Molpadia intermedia*	HM473932	CBG
BAM00070	DSPEC519-08	*Molpadia intermedia*	HM542278	CBG
BAM00071	DSPEC520-08	*Molpadia intermedia*	HM542279	CBG
HLC-23910	EQCS033-08	*Molpadia intermedia*	HM473931	CBG
HLC-L20101	ECNN102-08	*Ophiacantha bidentata*	HM543033	CBG
HLC-30779	ECNN154-09	*Ophiacantha bidentata*	HM400539	CBG
10CHEC-063	CHEC053-12	*Ophiacantha bidentata*	KU495769	CBG
HLC-30273	ECNN097-08	*Ophiacantha bidentata*	HM543032	CBG
HLC-L13502	ECNN093-08	*Ophiacantha bidentata*	GU670188	CBG
09CHON-038	CHONE038-10	*Ophiacantha bidentata*	HM405904	CBG
09CHON-022	CHONE022-10	*Ophiacantha bidentata*	HM405889	CBG
09CHON-021	CHONE021-10	*Ophiacantha bidentata*	HM405888	CBG
HLC-30043	ECNN014-08	*Ophiacantha bidentata*	HM543031	CBG
09CHON-030	CHONE030-10	*Ophiocten sericeum*	HM405897	CBG
09CHON-032	CHONE032-10	*Ophiocten sericeum*	HM405899	CBG
HLC-30778	ECNN153-09	*Ophiocten sericeum*	HM543039	CBG
HLC-30041	ECNN012-08	*Ophiocten sericeum*	HM543034	CBG
09CHON-027	CHONE027-10	*Ophiocten sericeum*	HM405894	CBG
HLC-30092	ECNN052-08	*Ophiocten sericeum*	HM543036	CBG
HLC-30530	ECNN095-08	*Ophiocten sericeum*	GU670187	CBG
09CHON-048	CHONE048-10	*Ophiocten sericeum*	HM405914	CBG
09CHON-046	CHONE046-10	*Ophiocten sericeum*	HM405912	CBG
09CHON-029	CHONE029-10	*Ophiocten sericeum*	HM405896	CBG
09CHON-044	CHONE044-10	*Ophiocten sericeum*	HM405910	CBG
09CHON-041	CHONE041-10	*Ophiocten sericeum*	HM405907	CBG
HLC-30525	ECNN099-08	*Ophiocten sericeum*	HM543037	CBG
10CHEC-013	CHEC013-11	*Ophiocten sericeum*	JN314244	CBG
09CHON-028	CHONE028-10	*Ophiocten sericeum*	HM405895	CBG
09CHON-042	CHONE042-10	*Ophiocten sericeum*	HM405908	CBG
HLC-30784	ECNN159-09	*Ophiocten sericeum*	HM543040	CBG
09CHON-031	CHONE031-10	*Ophiocten sericeum*	HM405898	CBG
09CHON-047	CHONE047-10	*Ophiocten sericeum*	HM405913	CBG
HLC-L20302	ECNN110-08	*Ophiocten sericeum*	HM543038	CBG
09CHON-045	CHONE045-10	*Ophiocten sericeum*	HM405911	CBG
HLC-30137	ECNN031-08	*Ophiocten sericeum*	HM543035	CBG
INV0653_03	ECNN118-08	*Ophiopholis aculeata*	HM473854	CBG
HUNT0059	DSPEC736-08	*Ophiopholis aculeata*	HM542297	CBG
HUNT0053	DSPEC730-08	*Ophiopholis aculeata*	HM542294	CBG
HUNT0057	DSPEC734-08	*Ophiopholis aculeata*	HM542295	CBG
CECE07-055	DSPEC125-07	*Ophiopholis aculeata*	HM542286	CBG
CECE07-056	DSPEC126-07	*Ophiopholis aculeata*	HM542287	CBG
CECE07-011	DSPEC081-07	*Ophiopholis aculeata*	HM542281	CBG
INV0641_01	ECNN114-08	*Ophiopholis aculeata*	HM473850	CBG
HUNT0075	DSPEC752-08	*Ophiopholis aculeata*	HM542298	CBG
HUNT0034	DSPEC711-08	*Ophiopholis aculeata*	HM542289	CBG

INV0653_01	ECNN116-08	*Ophiopholis aculeata*	HM473852	CBG
CECE07-054	DSPEC124-07	*Ophiopholis aculeata*	HM542280	CBG
09PROBE-02029	CCANN523-09	*Ophiopholis aculeata*	GU670181	CBG
08PROBE224_01	ECCH014-09	*Ophiopholis aculeata*	HM473856	CBG
CECE07-057	DSPEC127-07	*Ophiopholis aculeata*	HM542288	CBG
CECE07-012	DSPEC082-07	*Ophiopholis aculeata*	HM542282	CBG
HUNT0058	DSPEC735-08	*Ophiopholis aculeata*	HM542296	CBG
HUNT0052	DSPEC729-08	*Ophiopholis aculeata*	HM542293	CBG
HUNT0049	DSPEC726-08	*Ophiopholis aculeata*	HM542290	CBG
CECE07-013	DSPEC083-07	*Ophiopholis aculeata*	HM542283	CBG
INV0653_02	ECNN117-08	*Ophiopholis aculeata*	HM473853	CBG
HUNT0051	DSPEC728-08	*Ophiopholis aculeata*	HM542292	CBG
INV0653_04	ECNN119-08	*Ophiopholis aculeata*	HM473855	CBG
CECE07-015	DSPEC085-07	*Ophiopholis aculeata*	HM542285	CBG
CECE07-014	DSPEC084-07	*Ophiopholis aculeata*	HM542284	CBG
HUNT0050	DSPEC727-08	*Ophiopholis aculeata*	HM542291	CBG
INV0641_02	ECNN115-08	*Ophiopholis aculeata*	HM473851	CBG
HLC-24059	EQCS002-08	*Ophiopholis japonica*	HM473933	CBG
HLC-24060	EQCS003-08	*Ophiopholis japonica*	HM473934	CBG
10BIOBC-EC002	EQCS077-11	*Ophiopholis kennerlyi*	KU495831	CBG
BAM00138	DSPEC587-08	*Ophiopholis kennerlyi*	HM542306	CBG
BAM00040	DSPEC489-08	*Ophiopholis kennerlyi*	HM542300	CBG
BAM00039	DSPEC488-08	*Ophiopholis kennerlyi*	HM542299	CBG
10BIOBC-EC048	EQCS097-11	*Ophiopholis kennerlyi*	KU495807	CBG
BAM00094	DSPEC543-08	*Ophiopholis kennerlyi*	HM542301	CBG
10BIOBC-EC144	EQCS166-11	*Ophiopholis kennerlyi*	KU495756	CBG
10BIOBC-EC149	EQCS170-11	*Ophiopholis kennerlyi*	KU495761	CBG
BAM00137	DSPEC586-08	*Ophiopholis kennerlyi*	HM542305	CBG
10BIOBC-EC120	EQCS147-11	*Ophiopholis kennerlyi*	KU495900	CBG
BAM00130	DSPEC579-08	*Ophiopholis kennerlyi*	HM542302	CBG
10BIOBC-EC146	EQCS168-11	*Ophiopholis kennerlyi*	KU495798	CBG
10BIOBC-EC049	EQCS098-11	*Ophiopholis kennerlyi*	KU495862	CBG
10BIOBC-EC058	EQCS178-11	*Ophiopholis kennerlyi*	KU495781	CBG
BAM00135	DSPEC584-08	*Ophiopholis kennerlyi*	HM542304	CBG
10BIOBC-EC148	EQCS169-11	*Ophiopholis kennerlyi*	KU495763	CBG
10BIOBC-EC145	EQCS167-11	*Ophiopholis kennerlyi*	KU495808	CBG
10BIOBC-EC065	EQCS105-11	*Ophiopholis kennerlyi*	KU495856	CBG
10BIOBC-EC088	EQCS122-11	*Ophiopholis kennerlyi*	KU495794	CBG
BAM00131	DSPEC580-08	*Ophiopholis kennerlyi*	HM542303	CBG
10BIOBC-EC023	EQCS087-11	*Ophiopholis kennerlyi*	KU495789	CBG
HLC-24061	EQCS004-08	*Ophiopholis* sp. *AAE1685*	HM473937	CBG
HLC-24062	EQCS005-08	*Ophiopholis* sp. *AAE1685*	HM473938	CBG
HLC-24095	EQCS016-08	*Ophiopholis* sp. *AAE1685*	HM473936	CBG
HLC-24064*	EQCS007-08	*Ophiopholis* sp. *AAE1685*	HM473935	CBG
09CHON-023	CHONE023-10	*Ophiopleura borealis*	HM405890	CBG
09CHON-024	CHONE024-10	*Ophiopleura borealis*	HM405891	CBG
09CHON-025	CHONE025-10	*Ophiopleura borealis*	HM405892	CBG
09CHON-013	CHONE013-10	*Ophiopleura borealis*	HM405881	CBG
09CHON-015	CHONE015-10	*Ophiopleura borealis*	HM405883	CBG
09CHON-016	CHONE016-10	*Ophiopleura borealis*	HM405884	CBG
10CHEC-049	CHEC039-12	*Ophiopleura borealis*	KU495863	CBG
09CHON-014	CHONE014-10	*Ophiopleura borealis*	HM405882	CBG
09CHON-026	CHONE026-10	*Ophiopleura borealis*	HM405893	CBG
10CHEC-064	CHEC054-12	*Ophiopleura borealis*	KU495795	CBG
10CHEC-065	CHEC055-12	*Ophiopleura borealis*	KU495815	CBG
BIOUG14666-B09	ARCMI399-14	*Ophiopus* sp. *ACR1730*	KU495904	CBG
BIOUG14666-B10	ARCMI400-14	*Ophiopus* sp. *ACR1730*	KU495787	CBG
BAM00226	DSPEC675-08	*Ophiura luetkenii*	HM542310	CBG

BAM00228	DSPEC677-08	*Ophiura luetkenii*	HM542312	CBG
10BIOBC-EC075	EQCS109-11	*Ophiura luetkenii*	KU495745	CBG
10BIOBC-EC077	EQCS111-11	*Ophiura luetkenii*	KU495892	CBG
BAM00227	DSPEC676-08	*Ophiura luetkenii*	HM542311	CBG
10BIOBC-EC108	EQCS226-11	*Ophiura luetkenii*	KU495869	CBG
BAM00224	DSPEC673-08	*Ophiura luetkenii*	HM542308	CBG
10BIOBC-EC076	EQCS110-11	*Ophiura luetkenii*	KU495915	CBG
10BIOBC-EC107	EQCS138-11	*Ophiura luetkenii*	KU495766	CBG
10BIOBC-EC093	EQCS127-11	*Ophiura luetkenii*	KU495757	CBG
10BIOBC-EC106	EQCS137-11	*Ophiura luetkenii*	KU495758	CBG
10BIOBC-EC105	EQCS136-11	*Ophiura luetkenii*	KU495918	CBG
10BIOBC-EC109	EQCS227-11	*Ophiura luetkenii*	KU495916	CBG
BAM00225	DSPEC674-08	*Ophiura luetkenii*	HM542309	CBG
HUNT0055	DSPEC732-08	*Ophiura robusta*	HM542313	CBG
HLC-L20301	ECNN109-08	*Ophiura robusta*	GU670189	CBG
HLC-L20201	ECNN100-08	*Ophiura robusta*	HM543045	CBG
07PROBE-ECH018	DSPEC062-07	*Ophiura robusta*	HM473859	CBG
08PROBE242_02	ECCH017-09	*Ophiura robusta*	HM473872	CBG
09CHON-039	CHONE039-10	*Ophiura robusta*	HM405905	CBG
08PROBE242_01	ECCH016-09	*Ophiura robusta*	HM473871	CBG
HLC-30783	ECNN158-09	*Ophiura robusta*	HM543047	CBG
09CHON-040	CHONE040-10	*Ophiura robusta*	HM405906	CBG
09CHON-043	CHONE043-10	*Ophiura robusta*	HM405909	CBG
08PROBE189_01	ECCH001-09	*Ophiura robusta*	HM473863	CBG
HLC-30025	ECNN027-08	*Ophiura robusta*	HM543044	CBG
08PROBE254_02	ECCH011-09	*Ophiura robusta*	HM473869	CBG
07PROBE-ECH022	DSPEC066-07	*Ophiura robusta*	HM473862	CBG
08PROBE248_01	ECCH003-09	*Ophiura robusta*	HM473864	CBG
08PROBE248_02	ECCH004-09	*Ophiura robusta*	HM473865	CBG
HLC-L20202	ECNN101-08	*Ophiura robusta*	HM543046	CBG
08PROBE254_03	ECCH012-09	*Ophiura robusta*	HM473870	CBG
07PROBE-ECH011	DSPEC055-07	*Ophiura robusta*	HM473857	CBG
07PROBE-ECH012	DSPEC056-07	*Ophiura robusta*	HM473858	CBG
07PROBE-ECH020	DSPEC064-07	*Ophiura robusta*	HM473860	CBG
08PROBE230_01	ECCH008-09	*Ophiura robusta*	HM473867	CBG
07PROBE-ECH021	DSPEC065-07	*Ophiura robusta*	HM473861	CBG
08PROBE211_03	ECCH007-09	*Ophiura robusta*	HM473866	CBG
08PROBE254_01	ECCH010-09	*Ophiura robusta*	HM473868	CBG
10CHEC-050	CHEC040-12	*Ophiura sarsii*	KU495803	CBG
HLC-23972	EQCS044-08	*Ophiura sarsii*	GU670192	CBG
HLC-24014	EQCS056-08	*Ophiura sarsii*	HM473940	CBG
HLC-30089	ECNN021-08	*Ophiura sarsii*	HM543043	CBG
HLC-30064	ECNN011-08	*Ophiura sarsii*	HM543041	CBG
09CHON-018	CHONE018-10	*Ophiura sarsii*	HM405886	CBG
HLC-24096	EQCS017-08	*Ophiura sarsii*	HM473939	CBG
09CHON-019	CHONE019-10	*Ophiura sarsii*	HM405887	CBG
10CHEC-074	CHEC064-12	*Ophiura sarsii*	KU495767	CBG
10CHEC-054	CHEC044-12	*Ophiura sarsii*	KU495813	CBG
10CHEC-051	CHEC041-12	*Ophiura sarsii*	KU495764	CBG
09CHON-017	CHONE017-10	*Ophiura sarsii*	HM405885	CBG
HLC-30042	ECNN013-08	*Ophiura sarsii*	HM543042	CBG
10CHEC-053	CHEC043-12	*Ophiura sarsii*	KU495777	CBG
10CHEC-073	CHEC063-12	*Ophiura sarsii*	KU495897	CBG
10CHEC-062	CHEC052-12	*Ophiura sarsii*	KU495754	CBG
BAM00052	DSPEC501-08	*Orthasterias koehleri*	HM542317	CBG
BAM00074	DSPEC523-08	*Orthasterias koehleri*	HM542318	CBG
BAM00185	DSPEC634-08	*Orthasterias koehleri*	HM542316	CBG
BAM00182	DSPEC631-08	*Orthasterias koehleri*	HM542314	CBG

BAM00183	DSPEC632-08	*Orthasterias koehleri*	HM542315	CBG
HLC-23906	EQCS030-08	*Orthasterias koehleri*	GU670190	CBG
BAM00078	DSPEC527-08	*Parastichopus californicus*	HM542322	CBG
BAM00076	DSPEC525-08	*Parastichopus californicus*	HM542320	CBG
10BIOBC-EC071	EQCS197-11	*Parastichopus californicus*	KU495843	CBG
10BIOBC-EC064	EQCS104-11	*Parastichopus californicus*	KU495759	CBG
BAM00077	DSPEC526-08	*Parastichopus californicus*	HM542321	CBG
BAM00154	DSPEC603-08	*Parastichopus californicus*	HM542323	CBG
HLC-24072	EQCS012-08	*Pedicellaster magister*	HM473942	CBG
HLC-24071	EQCS011-08	*Pedicellaster magister*	HM473941	CBG
HLC-L14201	ECNN083-08	*Pentamera calcigera*	HM543058	CBG
HLC-L06403	ECNN068-08	*Pentamera calcigera*	HM543057	CBG
HLC-30032	ECNN025-08	*Pentamera calcigera*	HM543053	CBG
HLC-L13701	ECNN084-08	*Pentamera calcigera*	HM543059	CBG
HLC-L06401	ECNN066-08	*Pentamera calcigera*	HM543055	CBG
HLC-L06402	ECNN067-08	*Pentamera calcigera*	HM543056	CBG
HLC-30280	ECNN037-08	*Pentamera calcigera*	HM543054	CBG
09PROBE-02030	CCANN524-09	*Pentamera calcigera*	HM473873	CBG
BAM00031	DSPEC480-08	*Pentamera cf. pediparva*	HM542327	CBG
BAM00030	DSPEC479-08	*Pentamera cf. pediparva*	HM542326	CBG
BAM00072*	DSPEC521-08	*Pentamera cf. pseudocalcigera*	HM542325	CBG
HLC-24101*	EQCS019-08	*Pentamera cf. pseudocalcigera*	HM473944	CBG
HLC-24094*	EQCS015-08	*Pentamera cf. pseudocalcigera*	HM473943	CBG
BAM00171	DSPEC620-08	*Pisaster brevispinus*	HM542329	CBG
BAM00170	DSPEC619-08	*Pisaster brevispinus*	HM542328	CBG
10BIOBC-EC127	EQCS203-11	*Pisaster ochraceus*	KU495801	CBG
10BIOBC-EC007	EQCS210-11	*Pisaster ochraceus*	KU495790	CBG
10BIOBC-EC151	EQCS233-12	*Pisaster ochraceus*	KU495774	CBG
BAM00208	DSPEC657-08	*Pisaster ochraceus*	HM542336	CBG
BAM00212	DSPEC661-08	*Pisaster ochraceus*	HM542340	CBG
10BIOBC-EC063	EQCS182-11	*Pisaster ochraceus*	KU495751	CBG
BAM00211	DSPEC660-08	*Pisaster ochraceus*	HM542339	CBG
10BIOBC-EC062	EQCS181-11	*Pisaster ochraceus*	KU495821	CBG
HLC-23989	EQCS051-08	*Pisaster ochraceus*	HM473946	CBG
BAM00210	DSPEC659-08	*Pisaster ochraceus*	HM542338	CBG
HLC-23990	EQCS052-08	*Pisaster ochraceus*	HM473947	CBG
10BIOBC-EC061	EQCS103-11	*Pisaster ochraceus*	KU495906	CBG
BAM00209	DSPEC658-08	*Pisaster ochraceus*	HM542337	CBG
HLC-23988	EQCS050-08	*Pisaster ochraceus*	HM473945	CBG
10CHEC-014	CHEC014-11	*Pontaster tenuispinus*	JF884200	CBG
10CHEC-046	CHEC080-12	*Pontaster tenuispinus*	KU495872	CBG
10CHEC-045	CHEC079-12	*Pontaster tenuispinus*	KU495913	CBG
09CHON-036	CHONE036-10	*Pontaster tenuispinus*	HM405903	CBG
09CHON-033	CHONE033-10	*Pontaster tenuispinus*	HM405900	CBG
10CHEC-026	CHEC026-11	*Pontaster tenuispinus*	KU495868	CBG
09CHON-034	CHONE034-10	*Pontaster tenuispinus*	HM405901	CBG
09CHON-035	CHONE035-10	*Pontaster tenuispinus*	HM405902	CBG
10CHEC-044	CHEC078-12	*Pontaster tenuispinus*	KU495737	CBG
09CHON-012	CHONE012-10	*Psilaster andromeda*	HM405880	CBG
10CHEC-070	CHEC060-12	*Psilaster andromeda*	KU495817	CBG
10CHEC-040	CHEC074-12	*Psilaster andromeda*	KU495793	CBG
10CHEC-043	CHEC077-12	*Psilaster andromeda*	KU495775	CBG
BAM00145	DSPEC594-08	*Psolus chitonoides*	HM542343	CBG
BAM00038	DSPEC487-08	*Psolus chitonoides*	HM542341	CBG
BAM00146	DSPEC595-08	*Psolus chitonoides*	HM542344	CBG
BAM00144	DSPEC593-08	*Psolus chitonoides*	HM542342	CBG
CECE07-050	DSPEC120-07	*Psolus fabricii*	HM542348	CBG
CECE07-048	DSPEC118-07	*Psolus fabricii*	HM542347	CBG

09PROBE-01149	CCANN586-09	*Psolus fabricii*	HM405487	CBG
CECE07-051	DSPEC121-07	*Psolus fabricii*	HM542349	CBG
INV0663_02	ECNN125-08	*Psolus fabricii*	GU670175	CBG
07PROBE-ECH024	DSPEC068-07	*Psolus fabricii*	HM473874	CBG
INV0663_04	ECNN127-08	*Psolus fabricii*	GU670178	CBG
CECE07-047	DSPEC117-07	*Psolus fabricii*	HM542346	CBG
INV0663_01	ECNN124-08	*Psolus fabricii*	GU670173	CBG
HLC-30236	ECNN051-08	*Psolus fabricii*	HM543061	CBG
INV0663_03	ECNN126-08	*Psolus fabricii*	GU670179	CBG
INV0672_03	ECNN138-08	*Psolus fabricii*	GU670180	CBG
HLC-30254	ECNN024-08	*Psolus fabricii*	HM543060	CBG
INV0639_02	ECNN113-08	*Psolus fabricii*	GU670172	CBG
INV0639_01	ECNN112-08	*Psolus fabricii*	GU670171	CBG
CECE07-046	DSPEC116-07	*Psolus fabricii*	HM542345	CBG
HLC-30151	ECNN063-08	*Psolus phantapus*	HM543064	CBG
HLC-30255	ECNN069-08	*Psolus phantapus*	HM543065	CBG
HLC-L01301	ECNN070-08	*Psolus phantapus*	HM543066	CBG
HUNT0125	DSPEC803-09	*Psolus phantapus*	HM542351	CBG
HUNT0054	DSPEC731-08	*Psolus phantapus*	HM542350	CBG
HLC-L13301	ECNN055-08	*Psolus phantapus*	HM543062	CBG
HLC-L13302	ECNN056-08	*Psolus phantapus*	HM543063	CBG
HLC-24196	EQCS067-08	*Pteraster militaris*	HM473948	CBG
HUNT0039	DSPEC716-08	*Pteraster militaris*	HM542356	CBG
CECE07-005	DSPEC075-07	*Pteraster militaris*	HM542352	CBG
BAM00174	DSPEC623-08	*Pteraster militaris*	HM542355	CBG
BAM00173	DSPEC622-08	*Pteraster militaris*	HM542354	CBG
CECE07-028	DSPEC098-07	*Pteraster militaris*	HM542353	CBG
HUNT0056	DSPEC733-08	*Pteraster militaris*	HM542357	CBG
HUNT0133	DSPEC811-09	*Pteraster militaris*	GU670170	CBG
WSECH0022	WSEC022-09	*Pteraster militaris*	GU672429	CBG
WSECH0023	WSEC023-09	*Pteraster militaris*	GU672430	CBG
HLC-24067	EQCS057-08	*Pteraster* sp. *AAH7925*	HM473950	CBG
HLC-24104	EQCS021-08	*Pteraster* sp. *AAH7925*	HM473949	CBG
HLC-30404	ECNN047-08	*Pteraster* sp. *AAL2213*	HM543067	CBG
HLC-24172	EQCS029-08	*Pteraster tesselatus*	HM473951	CBG
BAM00054	DSPEC503-08	*Pteraster tesselatus*	HM542358	CBG
BAM00205	DSPEC654-08	*Pycnopodia helianthoides*	HM542362	CBG
BAM00206	DSPEC655-08	*Pycnopodia helianthoides*	HM542363	CBG
BAM00204	DSPEC653-08	*Pycnopodia helianthoides*	HM542361	CBG
10BIOBC-EC150	EQCS171-11	*Pycnopodia helianthoides*	KU495879	CBG
10BIOBC-EC018	EQCS213-11	*Pycnopodia helianthoides*	KU495875	CBG
BAM00203	DSPEC652-08	*Pycnopodia helianthoides*	HM542360	CBG
10BIOBC-EC043	EQCS172-11	*Pycnopodia helianthoides*	KU495762	CBG
10BIOBC-EC046	EQCS194-11	*Pycnopodia helianthoides*	KU495783	CBG
10BIOBC-EC047	EQCS177-11	*Pycnopodia helianthoides*	KU495772	CBG
BAM00207	DSPEC656-08	*Pycnopodia helianthoides*	HM542364	CBG
BAM00051	DSPEC500-08	*Solaster dawsoni*	HM542369	CBG
10BIOBC-EC045	EQCS193-11	*Solaster dawsoni*	KU495850	CBG
BAM00049	DSPEC498-08	*Solaster dawsoni*	HM542367	CBG
10BIOBC-EC010	EQCS081-11	*Solaster dawsoni*	KU495786	CBG
BAM00195	DSPEC644-08	*Solaster dawsoni*	HM542366	CBG
BAM00050	DSPEC499-08	*Solaster dawsoni*	HM542368	CBG
10BIOBC-EC054	EQCS175-11	*Solaster dawsoni*	KU495797	CBG
BAM00194	DSPEC643-08	*Solaster dawsoni*	HM542365	CBG
CECE07-035	DSPEC105-07	*Solaster endeca*	HM542373	CBG
CECE07-021	DSPEC091-07	*Solaster endeca*	HM542370	CBG
CECE07-022	DSPEC092-07	*Solaster endeca*	HM542371	CBG
HLC-30264	ECNN026-08	*Solaster endeca*	HM543068	CBG

BAM00189	DSPEC638-08	*Solaster endeca*	HM542375	CBG
CECE07-034	DSPEC104-07	*Solaster endeca*	HM542372	CBG
BAM00188	DSPEC637-08	*Solaster endeca*	HM542374	CBG
WSECH0014	WSEC014-09	*Solaster endeca*	GU672422	CBG
BAM00193	DSPEC642-08	*Solaster* sp. *AAF4823*	HM542377	CBG
BAM00187	DSPEC636-08	*Solaster* sp. *AAF4823*	HM542376	CBG
HLC-30096	ECNN004-08	*Solaster* sp. *AAF4824*	HM543069	CBG
10BIOBC-EC070	EQCS196-11	*Solaster stimpsoni*	KU495908	CBG
BAM00192	DSPEC641-08	*Solaster stimpsoni*	HM542380	CBG
BAM00057	DSPEC506-08	*Solaster stimpsoni*	HM542382	CBG
BAM00191	DSPEC640-08	*Solaster stimpsoni*	HM542379	CBG
BAM00190	DSPEC639-08	*Solaster stimpsoni*	HM542378	CBG
BAM00055	DSPEC504-08	*Solaster stimpsoni*	HM542381	CBG
HLC-L02802	ECNN106-08	*Stegophiura nodosa*	HM543050	CBG
HLC-30228	ECNN035-08	*Stegophiura nodosa*	HM543048	CBG
BIOUG14666-C02	ARCMI404-14	*Stegophiura nodosa*	KU495851	CBG
HLC-L02801	ECNN105-08	*Stegophiura nodosa*	HM543049	CBG
HLC-L02602	ECNN108-08	*Stegophiura nodosa*	HM543052	CBG
HLC-L02601	ECNN107-08	*Stegophiura nodosa*	HM543051	CBG
HLC-30330	ECNN042-08	*Stephanasterias albula*	HM543070	CBG
BAM00139	DSPEC588-08	*Strongylocentrotus droebachiensis*	HM542391	CBG
CECE07-058	DSPEC128-07	*Strongylocentrotus droebachiensis*	HM542384	CBG
CECE07-062	DSPEC132-07	*Strongylocentrotus droebachiensis*	HM542388	CBG
CECE07-063	DSPEC133-07	*Strongylocentrotus droebachiensis*	HM542389	CBG
CECE07-061	DSPEC131-07	*Strongylocentrotus droebachiensis*	HM542387	CBG
HUNT200	DSPEC835-11	*Strongylocentrotus droebachiensis*	KU495838	CBG
10BIOBC-EC006	EQCS209-11	*Strongylocentrotus droebachiensis*	KU495760	CBG
BAM00142	DSPEC591-08	*Strongylocentrotus droebachiensis*	HM542394	CBG
10BIOBC-EC025	EQCS216-11	*Strongylocentrotus droebachiensis*	KU495819	CBG
10BIOBC-00043	EQCS165-11	*Strongylocentrotus droebachiensis*	KU495871	CBG
CECE07-059	DSPEC129-07	*Strongylocentrotus droebachiensis*	HM542385	CBG
BAM00132	DSPEC581-08	*Strongylocentrotus droebachiensis*	HM542390	CBG
10BIOBC-EC024	EQCS088-11	*Strongylocentrotus droebachiensis*	KU495844	CBG
CECE07-060	DSPEC130-07	*Strongylocentrotus droebachiensis*	HM542386	CBG
BAM00143	DSPEC592-08	*Strongylocentrotus droebachiensis*	HM542395	CBG
BAM00141	DSPEC590-08	*Strongylocentrotus droebachiensis*	HM542393	CBG
BAM00140	DSPEC589-08	*Strongylocentrotus droebachiensis*	HM542392	CBG
HUNT201	DSPEC836-11	*Strongylocentrotus droebachiensis*	KU495802	CBG
HLC-23922	EQCS038-08	*Strongylocentrotus fragilis*	HM473953	CBG
HLC-23940	EQCS039-08	*Strongylocentrotus fragilis*	HM473954	CBG
HLC-24217	EQCS074-08	*Strongylocentrotus fragilis*	HM473956	CBG
HLC-23913	EQCS034-08	*Strongylocentrotus fragilis*	HM473952	CBG
HLC-23980	EQCS047-08	*Strongylocentrotus fragilis*	HM473955	CBG
INV0638_03	ECCH020-09	*Strongylocentrotus pallidus*	HM473886	CBG
HLC-24085	EQCS061-08	*Strongylocentrotus pallidus*	HM473957	CBG
10CHEC-008	CHEC008-11	*Strongylocentrotus pallidus*	KU495776	CBG
HLC-30466	ECNN098-08	*Strongylocentrotus pallidus*	HM543071	CBG
INV0638_05	ECCH022-09	*Strongylocentrotus pallidus*	HM473888	CBG
10CHEC-015	CHEC015-11	*Strongylocentrotus pallidus*	KU495746	CBG
INV0664_02	ECNN129-08	*Strongylocentrotus pallidus*	HM473878	CBG
INV0672_01	ECNN136-08	*Strongylocentrotus pallidus*	HM473879	CBG
INV0672_02	ECNN137-08	*Strongylocentrotus pallidus*	HM473880	CBG
INV0660	ECNN135-08	*Strongylocentrotus pallidus*	HM473876	CBG
INV0638_04	ECCH021-09	*Strongylocentrotus pallidus*	HM473887	CBG
INV0638_01	ECCH018-09	*Strongylocentrotus pallidus*	HM473884	CBG
INV0638_02	ECCH019-09	*Strongylocentrotus pallidus*	HM473885	CBG
INV0664_01	ECNN128-08	*Strongylocentrotus pallidus*	HM473877	CBG
10CHEC-016	CHEC016-11	*Strongylocentrotus pallidus*	KU495882	CBG

INV0673_03	ECNN145-08	*Strongylocentrotus pallidus*	HM473883	CBG
INV0638_06	ECCH023-09	*Strongylocentrotus pallidus*	HM473889	CBG
INV0673_02	ECNN144-08	*Strongylocentrotus pallidus*	HM473882	CBG
09CHON-003	CHONE003-10	*Strongylocentrotus pallidus*	HM405871	CBG
10CHEC-017	CHEC017-11	*Strongylocentrotus pallidus*	KU495873	CBG
09CHON-006	CHONE006-10	*Strongylocentrotus pallidus*	HM405874	CBG
09CHON-004	CHONE004-10	*Strongylocentrotus pallidus*	HM405872	CBG
09CHON-005	CHONE005-10	*Strongylocentrotus pallidus*	HM405873	CBG
INV0673_01	ECNN143-08	*Strongylocentrotus pallidus*	HM473881	CBG
10CHEC-009	CHEC009-11	*Strongylocentrotus pallidus*	KU495864	CBG
BAM00064	DSPEC513-08	*Strongylocentrotus purpuratus*	HM542408	CBG
10BIOBC-EC119	EQCS201-11	*Strongylocentrotus purpuratus*	KU495832	CBG
BAM00065	DSPEC514-08	*Strongylocentrotus purpuratus*	HM542409	CBG
BAM00062	DSPEC511-08	*Strongylocentrotus purpuratus*	HM542406	CBG
BAM00063	DSPEC512-08	*Strongylocentrotus purpuratus*	HM542407	CBG
BAM00066	DSPEC515-08	*Strongylocentrotus purpuratus*	HM542410	CBG
HLC-30005	ECNN008-08	*Strongylocentrotus* sp. *AAA9523*	HM543073	CBG
HLC-30001	ECNN007-08	*Strongylocentrotus* sp. *AAA9523*	HM543072	CBG
BAM00198	DSPEC647-08	*Stylasterias forreri*	HM542413	CBG
BAM00197	DSPEC646-08	*Stylasterias forreri*	HM542412	CBG
BAM00015	DSPEC464-08	*Stylasterias forreri*	HM542417	CBG
BAM00014	DSPEC463-08	*Stylasterias forreri*	HM542416	CBG
BAM00200	DSPEC649-08	*Stylasterias forreri*	HM542415	CBG
BAM00196	DSPEC645-08	*Stylasterias forreri*	HM542411	CBG
BAM00199	DSPEC648-08	*Stylasterias forreri*	HM542414	CBG
BAM00017	DSPEC466-08	*Stylasterias forreri*	HM542419	CBG
BAM00016	DSPEC465-08	*Stylasterias forreri*	HM542418	CBG
HUNT0073	DSPEC750-08	*Thyonidium drummondii*	HM400330	CBG
HUNT0064	DSPEC741-08	*Thyonidium drummondii*	HM400329	CBG
HUNT0144	DSPEC822-09	*Thyonidium drummondii*	HM400363	CBG
HUNT0143	DSPEC821-09	*Thyonidium drummondii*	HM400362	CBG
HUNT0074	DSPEC751-08	*Thyonidium drummondii*	HM542420	CBG
09CHON-011	CHONE011-10	*Urasterias lincki*	HM405879	CBG
BAM00229	DSPEC755-08	*Ophiopholis kennerlyi*	HM542307	CBG
RBCM EC00072	DSPEC205-08	*Ampheraster marianus*	HM542909	RBCM
RBCM EC00071	DSPEC204-08	*Ampheraster marianus*	HM542908	RBCM
RBCM EC00234	DSPEC367-08	*Amphiophiura superba*	HM400325	RBCM
RBCM EC00236	DSPEC369-08	*Amphiophiura superba*	HM400326	RBCM
RBCM EC00233	DSPEC366-08	*Amphiophiura superba*	HM400324	RBCM
RBCM EC00237	DSPEC370-08	*Amphiophiura superba*	HM400327	RBCM
RBCM EC00248	DSPEC381-08	*Asteronyx loveni*	HM542914	RBCM
RBCM EC00246	DSPEC379-08	*Asteronyx loveni*	HM542912	RBCM
RBCM EC00249	DSPEC382-08	*Asteronyx loveni*	HM542915	RBCM
RBCM EC00243	DSPEC376-08	*Asteronyx loveni*	HM542910	RBCM
RBCM EC00247	DSPEC380-08	*Asteronyx loveni*	HM542913	RBCM
RBCM EC00244	DSPEC377-08	*Asteronyx loveni*	HM542911	RBCM
RBCM EC00271	DSPEC404-08	*Asteroschema sublaeve*	HM400328	RBCM
RBCM EC00052	DSPEC185-08	*Benthopecten acanthonotus*	HM542921	RBCM
RBCM EC00050	DSPEC183-08	*Benthopecten acanthonotus*	HM542919	RBCM
RBCM EC00048	DSPEC181-08	*Benthopecten acanthonotus*	HM542917	RBCM
RBCM EC00051	DSPEC184-08	*Benthopecten acanthonotus*	HM542920	RBCM
RBCM EC00049	DSPEC182-08	*Benthopecten acanthonotus*	HM542918	RBCM
RBCM EC00047	DSPEC180-08	*Benthopecten acanthonotus*	HM542916	RBCM
RBCM EC00054	DSPEC187-08	*Benthopecten claviger*	HM542923	RBCM
RBCM EC00055	DSPEC188-08	*Benthopecten claviger*	HM542924	RBCM
RBCM EC00053	DSPEC186-08	*Benthopecten claviger*	HM542922	RBCM
NEOCAL07-0098	DSPEC004-07	*Brisaster latifrons*	HM542110	RBCM
NEOCAL07-0111	DSPEC017-07	*Brisaster latifrons*	HM542114	RBCM

NEOCAL07-0109	DSPEC015-07	*Brisaster latifrons*	HM542112	RBCM
NEOCAL07-0110	DSPEC016-07	*Brisaster latifrons*	HM542113	RBCM
NEOCAL07-0107	DSPEC013-07	*Brisaster latifrons*	HM542111	RBCM
NEOCAL07-0128	DSPEC034-07	*Ceramaster patagonicus*	HM542119	RBCM
NEOCAL07-0097	DSPEC003-07	*Ceramaster patagonicus*	HM542118	RBCM
NEOCAL07-0103	DSPEC009-07	*Ceramaster* sp. *AAI7443*	HM542117	RBCM
NEOCAL07-0102	DSPEC008-07	*Ceramaster* sp. *AAI7443*	HM542116	RBCM
RBCM EC00007	DSPEC140-08	*Crossaster borealis*	HM542925	RBCM
NEOCAL07-0118	DSPEC024-07	*Ctenodiscus crispatus*	HM542138	RBCM
NEOCAL07-0120	DSPEC026-07	*Ctenodiscus crispatus*	HM542140	RBCM
NEOCAL07-0108	DSPEC014-07	*Ctenodiscus crispatus*	HM542136	RBCM
NEOCAL07-0119	DSPEC025-07	*Ctenodiscus crispatus*	HM542139	RBCM
NEOCAL07-0117	DSPEC023-07	*Ctenodiscus crispatus*	HM542137	RBCM
RBCM EC00073	DSPEC206-08	*Diplopteraster multipes*	HM542926	RBCM
RBCM EC00025	DSPEC158-08	*Dipsacaster borealis*	HM400306	RBCM
RBCM EC00036	DSPEC169-08	*Eremicaster pacificus*	HM542930	RBCM
RBCM EC00028	DSPEC161-08	*Eremicaster pacificus*	HM542927	RBCM
RBCM EC00029	DSPEC162-08	*Eremicaster pacificus*	HM542928	RBCM
RBCM EC00035	DSPEC168-08	*Eremicaster pacificus*	HM542929	RBCM
NEOCAL07-0138	DSPEC044-07	*Evasterias troscheli*	HM542180	RBCM
RBCM EC00154	DSPEC287-08	*Freyellaster fecundus*	HM400321	RBCM
NEOCAL07-0095	DSPEC001-07	*Henricia aspera*	HM542213	RBCM
NEOCAL07-0131	DSPEC037-07	*Henricia* sp. *AAI1826*	HM400305	RBCM
RBCM EC00008	DSPEC141-08	*Heterozonias alternatus*	HM542931	RBCM
RBCM EC00124	DSPEC257-08	*Hippasteria californica*	HM400314	RBCM
RBCM EC00127	DSPEC260-08	*Hippasteria californica*	HM400316	RBCM
RBCM EC00122	DSPEC255-08	*Hippasteria californica*	HM400313	RBCM
RBCM EC00125	DSPEC258-08	*Hippasteria californica*	HM400315	RBCM
RBCM EC00027	DSPEC160-08	*Hymenodiscus pannychia*	HM542932	RBCM
RBCM EC00024	DSPEC157-08	*Leptychaster anomalus*	HM542933	RBCM
RBCM EC00165	DSPEC298-08	*Lophaster furcilliger*	HM542934	RBCM
NEOCAL07-0105	DSPEC011-07	*Lophaster furcilliger*	HM542254	RBCM
RBCM EC00168	DSPEC301-08	*Lophaster furcilliger*	HM542937	RBCM
RBCM EC00170	DSPEC303-08	*Lophaster furcilliger*	HM542939	RBCM
RBCM EC00167	DSPEC300-08	*Lophaster furcilliger*	HM542936	RBCM
RBCM EC00166	DSPEC299-08	*Lophaster furcilliger*	HM542935	RBCM
RBCM EC00169	DSPEC302-08	*Lophaster furcilliger*	HM542938	RBCM
NEOCAL07-0112	DSPEC018-07	*Luidia foliolata*	HM542255	RBCM
NEOCAL07-0121	DSPEC027-07	*Luidia foliolata*	HM542259	RBCM
NEOCAL07-0114	DSPEC020-07	*Luidia foliolata*	HM542257	RBCM
NEOCAL07-0113	DSPEC019-07	*Luidia foliolata*	HM542256	RBCM
NEOCAL07-0115	DSPEC021-07	*Luidia foliolata*	HM542258	RBCM
NEOCAL07-0106	DSPEC012-07	*Mediaster aequalis*	HM542263	RBCM
NEOCAL07-0100	DSPEC006-07	*Mediaster aequalis*	HM542261	RBCM
NEOCAL07-0099	DSPEC005-07	*Mediaster aequalis*	HM542260	RBCM
NEOCAL07-0101	DSPEC007-07	*Mediaster aequalis*	HM542262	RBCM
NEOCAL07-0124	DSPEC030-07	*Molpadia intermedia*	HM542275	RBCM
NEOCAL07-0125	DSPEC031-07	*Molpadia intermedia*	HM542276	RBCM
NEOCAL07-0122	DSPEC028-07	*Molpadia intermedia*	HM542273	RBCM
NEOCAL07-0116	DSPEC022-07	*Molpadia intermedia*	HM542272	RBCM
NEOCAL07-0123	DSPEC029-07	*Molpadia intermedia*	HM542274	RBCM
RBCM EC00098	DSPEC231-08	*Nearchaster aciculosus*	HM400307	RBCM
RBCM EC00198	DSPEC331-08	*Ophiocten hastatum*	HM542941	RBCM
RBCM EC00199	DSPEC332-08	*Ophiocten hastatum*	HM542942	RBCM
RBCM EC00197	DSPEC330-08	*Ophiocten hastatum*	HM542940	RBCM
RBCM EC00200	DSPEC333-08	*Ophiocten hastatum*	HM542943	RBCM
RBCM EC00215	DSPEC348-08	*Ophiomusium glabrum*	HM400322	RBCM
RBCM EC00217	DSPEC350-08	*Ophiomusium glabrum*	HM400323	RBCM

RBCM EC00226*	DSPEC359-08	*Ophiomusium lymani*	HM542944	RBCM
RBCM EC00228	DSPEC361-08	*Ophiopholis* sp. *AAE1685*	HM542945	RBCM
RBCM EC00208	DSPEC341-08	*Ophiophthalmus cataleimmoidus*	HM542946	RBCM
RBCM EC00188	DSPEC321-08	*Ophiophthalmus normani*	HM542948	RBCM
RBCM EC00191	DSPEC324-08	*Ophiophthalmus normani*	HM542949	RBCM
RBCM EC00186	DSPEC319-08	*Ophiophthalmus normani*	HM542947	RBCM
RBCM EC00183	DSPEC316-08	*Ophioscolex corynetes*	HM542951	RBCM
RBCM EC00181	DSPEC314-08	*Ophioscolex corynetes*	HM542950	RBCM
RBCM EC00210	DSPEC343-08	*Ophiosphalma jolliense*	HM542952	RBCM
RBCM EC00213	DSPEC346-08	*Ophiosphalma jolliense*	HM542955	RBCM
RBCM EC00214	DSPEC347-08	*Ophiosphalma jolliense*	HM542956	RBCM
RBCM EC00211	DSPEC344-08	*Ophiosphalma jolliense*	HM542953	RBCM
RBCM EC00212	DSPEC345-08	*Ophiosphalma jolliense*	HM542954	RBCM
RBCM EC00254	DSPEC387-08	*Ophiura luetkenii*	HM542957	RBCM
RBCM EC00304	DSPEC437-08	*Pannychia moseleyi*	HM542958	RBCM
NEOCAL07-0126	DSPEC032-07	*Parastichopus californicus*	HM542319	RBCM
RBCM EC00102	DSPEC235-08	*Pectinaster agassizi*	HM400309	RBCM
RBCM EC00101	DSPEC234-08	*Pectinaster agassizi*	HM400308	RBCM
RBCM EC00104	DSPEC237-08	*Pectinaster agassizi*	HM400310	RBCM
NEOCAL07-0130*	DSPEC036-07	*Pentamera cf. pseudocalcigera*	HM542324	RBCM
NEOCAL07-0137	DSPEC043-07	*Pisaster ochraceus*	HM542335	RBCM
NEOCAL07-0134	DSPEC040-07	*Pisaster ochraceus*	HM542332	RBCM
NEOCAL07-0136	DSPEC042-07	*Pisaster ochraceus*	HM542334	RBCM
NEOCAL07-0133	DSPEC039-07	*Pisaster ochraceus*	HM542331	RBCM
NEOCAL07-0132	DSPEC038-07	*Pisaster ochraceus*	HM542330	RBCM
NEOCAL07-0135	DSPEC041-07	*Pisaster ochraceus*	HM542333	RBCM
RBCM EC00119	DSPEC252-08	*Pseudarchaster dissonus*	HM400311	RBCM
RBCM EC00120	DSPEC253-08	*Pseudarchaster dissonus*	HM400312	RBCM
RBCM EC00130	DSPEC263-08	*Pseudarchaster dissonus*	HM400317	RBCM
RBCM EC00133	DSPEC266-08	*Pseudarchaster dissonus*	HM400319	RBCM
RBCM EC00131	DSPEC264-08	*Pseudarchaster dissonus*	HM400318	RBCM
RBCM EC00134	DSPEC267-08	*Pseudarchaster dissonus*	HM400320	RBCM
RBCM EC00144	DSPEC277-08	*Pseudarchaster parelii*	HM542959	RBCM
RBCM EC00146	DSPEC279-08	*Pseudarchaster parelii alascensis*	HM542964	RBCM
RBCM EC00140	DSPEC273-08	*Pseudarchaster parelii alascensis*	HM542960	RBCM
RBCM EC00143	DSPEC276-08	*Pseudarchaster parelii alascensis*	HM542962	RBCM
RBCM EC00147	DSPEC280-08	*Pseudarchaster parelii alascensis*	HM542965	RBCM
RBCM EC00148	DSPEC281-08	*Pseudarchaster parelii alascensis*	HM542966	RBCM
RBCM EC00142	DSPEC275-08	*Pseudarchaster parelii alascensis*	HM542961	RBCM
RBCM EC00145	DSPEC278-08	*Pseudarchaster parelii alascensis*	HM542963	RBCM
RBCM EC00290	DSPEC423-08	*Pseudostichopus mollis*	HM542967	RBCM
RBCM EC00291	DSPEC424-08	*Pseudostichopus mollis*	HM542968	RBCM
RBCM EC00288*	DSPEC421-08	*Pseudostichopus tuberosus*	HM542970	RBCM
RBCM EC00287*	DSPEC420-08	*Pseudostichopus tuberosus*	HM542969	RBCM
RBCM EC00038	DSPEC171-08	*Psilaster pectinatus*	HM542971	RBCM
RBCM EC00039	DSPEC172-08	*Psilaster pectinatus*	HM542972	RBCM
RBCM EC00040	DSPEC173-08	*Psilaster pectinatus*	HM542973	RBCM
RBCM EC00044	DSPEC177-08	*Psilaster pectinatus*	HM542975	RBCM
RBCM EC00043	DSPEC176-08	*Psilaster pectinatus*	HM542974	RBCM
RBCM EC00015	DSPEC148-08	*Pteraster coscinopeplus*	HM542976	RBCM
RBCM EC00011	DSPEC144-08	*Pteraster jordani*	HM542978	RBCM
RBCM EC00010	DSPEC143-08	*Pteraster jordani*	HM542977	RBCM
RBCM EC00012	DSPEC145-08	*Pteraster jordani*	HM542979	RBCM
NEOCAL07-0127	DSPEC033-07	*Pycnopodia helianthoides*	HM542359	RBCM
RBCM EC00075	DSPEC208-08	*Sagenaster evermanni*	HM542980	RBCM
RBCM EC00076	DSPEC209-08	*Sagenaster evermanni*	HM542981	RBCM
RBCM EC00086	DSPEC219-08	*Sagenaster evermanni*	HM542985	RBCM
RBCM EC00083	DSPEC216-08	*Sagenaster evermanni*	HM542983	RBCM

RBCM EC00084	DSPEC217-08	*Sagenaster evermanni*	HM542984	RBCM
RBCM EC00077	DSPEC210-08	*Sagenaster evermanni*	HM542982	RBCM
RBCM EC00006	DSPEC139-08	*Solaster paxillatus*	HM542992	RBCM
RBCM EC00001	DSPEC134-08	*Solaster paxillatus*	HM542987	RBCM
RBCM EC00003	DSPEC136-08	*Solaster paxillatus*	HM542989	RBCM
RBCM EC00005	DSPEC138-08	*Solaster paxillatus*	HM542991	RBCM
RBCM EC00018	DSPEC151-08	*Solaster paxillatus*	HM542986	RBCM
RBCM EC00002	DSPEC135-08	*Solaster paxillatus*	HM542988	RBCM
RBCM EC00004	DSPEC137-08	*Solaster paxillatus*	HM542990	RBCM
RBCM EC00277	DSPEC410-08	*Sperosoma biseriatum*	HM542993	RBCM
RBCM EC00258	DSPEC391-08	*Stegophiura carinata*	HM542994	RBCM
NEOCAL07-0129	DSPEC035-07	*Strongylocentrotus droebachiensis*	HM542383	RBCM
RBCM EC00045	DSPEC178-08	*Thrissacanthias penicillatus*	HM542995	RBCM
RBCM EC00060	DSPEC193-08	*Zoroaster ophiurus*	HM542997	RBCM
RBCM EC00062	DSPEC195-08	*Zoroaster ophiurus*	HM542999	RBCM
RBCM EC00064	DSPEC197-08	*Zoroaster ophiurus*	HM543001	RBCM
RBCM EC00063	DSPEC196-08	*Zoroaster ophiurus*	HM543000	RBCM
RBCM EC00061	DSPEC194-08	*Zoroaster ophiurus*	HM542998	RBCM
RBCM EC00059	DSPEC192-08	*Zoroaster ophiurus*	HM542996	RBCM
