# Supplementary material for: Exploring Canadian Echinoderm Diversity through DNA Barcodes
Source: PLoS One. 2016 Nov 21;11(11):e0166118. doi: 10.1371/journal.pone.0166118 (PMC5117606; doi:10.1371/journal.pone.0166118)
Supplement: S2 Table — List of 113 species with two or more individuals and their corresponding mean and maximum intraspecific distances (%K2P). (PDF) [file pone.0166118.s002.pdf]

Species	N	Mean Intraspecific Divergence (%K2P)	Max Intraspecific Divergence (%K2P)
Ampheraster marianus	2	0.15	0.15
Amphiodia cf. urtica	6	0.8	1.45
Amphiodia occidentalis	2	0	0
Amphiophiura superba	4	0.33	0.61
Amphioplus macraspis	6	0	0
Amphipholis sp. *AAD7203*	11	0.75	1.24
Amphipholis sp. *AAJ2302*	2	0	0
Amphipholis squamata	5	0	0
Asterias forbesi	10	0.34	0.62
Asterias rubens	11	0.06	0.33
Asterina miniata	14	0.33	0.94
Asteronyx loveni	6	0.34	0.92
Benthopecten acanthonotus	6	0.56	0.93
Benthopecten claviger	3	0.1	0.15
Brisaster latifrons	6	0	0
Ceramaster patagonicus	4	0.16	0.32
Ceramaster sp. *AAI7443*	2	0.31	0.31
Chiridota laevis	6	0	0
Crossaster papposus	21	0.49	1.24
Ctenodiscus crispatus	25	0.16	0.62
Cucumaria cf. lubrica	3	0.1	0.15
Cucumaria frondosa	18	0.43	0.98
Cucumaria miniata	11	0.63	1.71
Cucumaria pallida	4	0.88	1.4
Cucumaria pseudocurata	8	0.08	0.31
Dendraster excentricus	5	0.38	0.92
Dermasterias imbricata	13	0.34	0.79
Echinarachnius parma	5	0.06	0.16
Eremicaster pacificus	4	0.1	0.15
Eupentacta quinquesemita	4	0.31	0.46
Evasterias troscheli	14	0.18	0.63
Florometra serratissima	21	0.31	0.8
Gorgonocephalus arcticus	16	1.27	2.53
Gorgonocephalus eucnemis	4	0.48	0.96
Henricia oculata	7	0.68	1.26
Henricia sanguinolenta	2	0.39	0.39
Henricia sp. *AAB3569*	24	0.23	0.66
Henricia sp. *AAB9183*	16	0.62	1.55
Henricia sp. *AAD3482*	7	0.35	0.77
Henricia sp. *AAF2468*	6	0.14	0.31
Henricia sp. *AAF2496*	3	0.2	0.31
Henricia sp. *AAI1811*	8	0.07	0.16
Henricia sp. *AAI1812*	3	0.41	0.62
Henricia sp. *AAI1816*	3	0.1	0.15
Henricia sp. *AAI1817*	2	0.15	0.15
Henricia sp. *AAI6792*	2	0.15	0.15
Hippasteria californica	4	0.93	1.87
Hymenaster pellucidus	2	0	0
Leptasterias hexactis	28	1.54	3.62
Leptasterias littoralis	27	0.58	1.29
Leptasterias polaris	33	0.01	0.16
Leptosynapta clarki	10	0.56	2.67
Leptychaster pacificus	2	0.31	0.31
Lophaster furcilliger	9	1.43	2.33
Luidia foliolata	12	0.67	1.39
Mediaster aequalis	22	0.14	0.64
Mesocentrotus franciscanus	14	0.15	0.48
Molpadia intermedia	10	0.32	0.8
Ophiacantha bidentata	9	1.01	1.87
Ophiocten hastatum	4	0.18	0.32
Ophiocten sericeum	22	0.13	0.32

Ophiomusium glabrum	2	2.02	2.02
Ophiopholis aculeata	27	0.99	2.18
Ophiopholis japonica	2	0	0
Ophiopholis kennerlyi	22	0.67	2.02
Ophiopholis sp. *AAE1685*	5	2.26	3.45
Ophiophthalmus normani	3	0.59	0.67
Ophiopleura borealis	11	0.39	0.77
Ophiopus sp. *ACR1730*	2	0	0
Ophioscolex corynetes	2	0.31	0.31
Ophiosphalma jolliense	5	1.26	2.18
Ophiura luetkenii	15	0.31	0.93
Ophiura robusta	25	0.09	0.31
Ophiura sarsii	16	3.73	7.58
Orthasterias koehleri	6	0.26	0.62
Parastichopus californicus	7	0.22	0.62
Pectinaster agassizi	3	0.41	0.61
Pedicellaster magister	2	0	0
Pentamera calcigera	8	0.08	0.31
Pentamera cf. pediparva	2	0	0
Pentamera cf. pseudocalcigera	4	0.22	0.43
Pisaster brevispinus	2	0.33	0.33
Pisaster ochraceus	20	0.2	0.8
Pontaster tenuispinus	9	0.3	0.61
Pseudarchaster dissonus	6	0.8	1.23
Pseudarchaster parelii alascensis	7	0.29	0.62
Pseudostichopus mollis	2	0	0
Pseudostichopus tuberosus	2	0.22	0.22
Psilaster andromeda	4	0.2	0.31
Psilaster pectinatus	5	0.25	0.47
Psolus chitonoides	4	0.15	0.23
Psolus fabricii	16	0.06	0.43
Psolus phantapus	7	0.24	0.51
Pteraster jordani	3	0.2	0.31
Pteraster militaris	8	1.67	2.98
Pteraster sp. *AAH7925*	2	0.61	0.61
Pteraster tesselatus	2	0.15	0.15
Pycnopodia helianthoides	11	0.33	0.94
Sagenaster evermanni	6	0.09	0.46
Solaster dawsoni	8	0.3	0.63
Solaster endeca	7	0.88	2.02
Solaster paxillatus	7	0.38	0.77
Solaster sp. *AAF4823*	2	0	0
Solaster stimpsoni	6	0.23	0.46
Stegophiura nodosa	6	0	0
Strongylocentrotus droebachiensis	19	0.18	0.53
Strongylocentrotus fragilis	5	0.11	0.31
Strongylocentrotus pallidus	25	0.05	0.31
Strongylocentrotus purpuratus	6	0.35	0.77
Strongylocentrotus sp. *AAA9523*	2	0.77	0.77
Stylasterias forreri	9	0.06	0.16
Thyonidium drummondii	5	0.64	1.35
Zoroaster ophiurus	6	0.18	0.31
