# Supplementary material for: Exploring Canadian Echinoderm Diversity through DNA Barcodes
Source: PLoS One. 2016 Nov 21;11(11):e0166118. doi: 10.1371/journal.pone.0166118 (PMC5117606; doi:10.1371/journal.pone.0166118)
Supplement: S3 Table — List of BINs with corresponding species ID, number of individuals, and locality information. Localities from a particular province/territory are separated by a comma, while semi-colon separates those from another province/territory. Species with multiple BIN assignments are in BOLD. Locality codes are as follows: Alutasivik Island (AI), Amundsen Gulf (AG), Baffin Bay (BB), Bamfield (BA), Barkley Canyon (BC), Barkley Sound (BS), Beaufort Sea (BE), Cape Scott (CS), Cape St. James (CJ), Churchill (CH), Cornwallis Island (CI), Devon Island (DI), Durban Harbour (DH), Haida Gwaii (HG), Howe Sound (HS), Hudson Bay (HB), Igloolik (IG), Kyoquot Sound (KS), Labrador Sea (LS), Lancaster Sound (LA), Nanaimo (NA), Nootka Sound (NS), Prince of Wales Strait (PW), Qikiqtarjuac (QK), Quatsino Sound (QS), Resolute (RE), Sechelt (SE), Somerset Island (SI), St. Andrews (SA), Vancouver Island (VI). *sequences lacking a BIN ID. (PDF) [file pone.0166118.s003.pdf]

| BIN | Species | N | Locality | | |
|---|---|---|---|---|---|
| | | | Pacific | Arctic | Atlantic |
| BOLD:AAJ2284 | *Ampheraster marianus* | 2 | NS | | |
| BOLD:AAF4324 | *Amphiodia cf. urtica* | 6 | BA; HG | | |
| BOLD:AAU6175 | *Amphiodia occidentalis* | 2 | HG | | |
| BOLD:AAE6089 | *Amphiophiura superba* | 4 | CS; HG | | |
| BOLD:AAD7270 | *Amphioplus macraspis* | 6 | BA | | |
| BOLD:AAD7203 | *Amphipholis* sp. *AAD7203* | 11 | BA; SE | | |
| BOLD:AAJ2302 | *Amphipholis* sp. *AAJ2302* | 2 | BA | | |
| BOLD:AAU6176 | *Amphipholis* sp. *AAU6176* | 1 | BA | | |
| BOLD:AAE2589 | *Amphipholis squamata* | 5 | | | SA |
| BOLD:AAB6163 | *Asterias forbesi* | 10 | | | SA |
| BOLD:AAB8222 | *Asterias rubens* | 11 | | | SA |
| BOLD:AAD9834 | *Asterina miniata* | 14 | BA; HG | | |
| BOLD:AAD8519 | *Asteronyx loveni* | 6 | BC; KS; NS | | |
| BOLD:AAJ9638 | *Asteroschema sublaeve* | 1 | HG | | |
| BOLD:AAD5744 | *Benthopecten acanthonotus* | 6 | HG; QS | | |
| BOLD:AAF7746 | *Benthopecten claviger* | 3 | CS; KS | | |
| BOLD:AAD7666 | *Brisaster latifrons* | 6 | BA; HS | | |
| BOLD:AAE9849 | *Ceramaster patagonicus* | 4 | HS; NA | | |
| BOLD:AAI7443 | *Ceramaster* sp. *AAI7443* | 2 | HS | | |
| BOLD:AAE2232 | *Chiridota laevis* | 6 | | | AI; SA |
| BOLD:AAR4792 | *Crossaster borealis* | 1 | CJ | | |
| BOLD:AAB5053 | *Crossaster papposus* | 21 | BA; HG; NA | RE; DI ; CH | SA |
| BOLD:AAC2685 | *Ctenodiscus crispatus* | 25 | HG; HS | BB; BE; HB; PW | |
| BOLD:AAE7780 | *Cucumaria cf. lubrica* | 3 | BA | | |
| BOLD:AAA1587 | *Cucumaria frondosa* | 18 | | IG; RE; SI | AI; SA |
| BOLD:AAC2853 | *Cucumaria miniata* | 11 | BA; HG | | |
| BOLD:AAE3243 | *Cucumaria pallida* | 4 | BA; NA | | |
| BOLD:AAJ5797 | *Cucumaria piperata* | 1 | BA | | |
| BOLD:AAJ5805 | *Cucumaria pseudocurata* | 8 | BA | | |
| BOLD:AAE3525 | *Dendraster excentricus* | 5 | NA | | |

| | | | | | |
|---|---|---|---|---|---|
| BOLD:AAD0458 | *Dermasterias imbricata* | 13 | BA; HG; NA | | |
| BOLD:AAX9511 | *Diplopteraster multipes* | 1 | BS | | |
| BOLD:AAL1960 | *Dipsacaster borealis* | 1 | HG | | |
| BOLD:AAE0410 | *Echinarachnius parma* | 5 | | | SA |
| BOLD:AAG3760 | *Ekmania barthii* | 1 | | CH | |
| BOLD:AAF0037 | *Eremicaster pacificus* | 4 | QS; VI | | |
| BOLD:AAD9632 | *Eupentacta quinquesemita* | 4 | BA | | |
| BOLD:AAD1220 | *Evasterias troscheli* | 14 | BA; HG; HS | | |
| BOLD:AAC3353 | *Florometra serratissima* | 21 | | BB; BE; IG | |
| BOLD:AAL2790 | *Freyellaster fecundus* | 1 | VI | | |
| **BOLD:AAC8875** | ***Gorgonocephalus arcticus*** | **7** | | **BB; RE** | **SA** |
| **BOLD:ABY6858** | ***Gorgonocephalus arcticus*** | **9** | | **BB; RE** | |
| BOLD:AAE7308 | *Gorgonocephalus eucnemis* | 4 | HG; NA | | |
| BOLD:AAI1835 | *Henricia aspera* | 1 | HS | | |
| BOLD:AAE3892 | *Henricia leviuscula spiculifera* | 1 | HG | | |
| BOLD:AAD3463 | *Henricia oculata* | 7 | HG | | SA |
| BOLD:AAF2495 | *Henricia sanguinolenta* | 2 | | | SA |
| BOLD:AAB3569 | *Henricia* sp. *AAB3569* | 24 | | | SA |
| BOLD:AAB9183 | *Henricia* sp. *AAB9183* | 16 | HG | BE | SA |
| BOLD:AAD3482 | *Henricia* sp. *AAD3482* | 7 | | IG | SA |
| BOLD:AAF2468 | *Henricia* sp. *AAF2468* | 6 | BA; SE | | |
| BOLD:AAF2496 | *Henricia* sp. *AAF2496* | 3 | BA; NA | | |
| BOLD:AAI1811 | *Henricia* sp. *AAI1811* | 8 | BA; HG | | |
| BOLD:AAI1812 | *Henricia* sp. *AAI1812* | 3 | BA; HG | | |
| BOLD:AAI1815 | *Henricia* sp. *AAI1815* | 1 | HG | | |
| BOLD:AAI1816 | *Henricia* sp. *AAI1816* | 3 | BA | | |
| BOLD:AAI1817 | *Henricia* sp. *AAI1817* | 2 | BA | | |
| BOLD:AAI1826 | *Henricia* sp. *AAI1826* | 1 | HS | | |
| BOLD:AAI6792 | *Henricia* sp. *AAI6792* | 2 | NA | | |
| BOLD:AAX0858 | *Heterozonias alternatus* | 1 | CJ | | |
| BOLD:AAE4144 | *Hippasteria californica* | 4 | BC; HG | | |
| BOLD:AAU5076 | *Hymenaster pellucidus* | 2 | | BB | |
| BOLD:AAX2049 | *Hymenodiscus pannychia* | 1 | VI | | |
| **BOLD:AAA8519** | ***Leptasterias hexactis*** | **7** | **BA; HG** | | |

| | | | | | |
|---|---|---|---|---|---|
| **BOLD:AAM1484** | ***Leptasterias hexactis*** | **21** | **BA; HG** | | |
| BOLD:AAB2709 | *Leptasterias littoralis* | 27 | | CH ; DI; RE | |
| BOLD:ACE6383 | *Leptasterias polaris* | 33 | | CH ; RE | |
| **BOLD:AAC6788** | ***Leptosynapta clarki*** | **9** | **BA** | | |
| **BOLD:AAC6789** | ***Leptosynapta clarki*** | **1** | **BA** | | |
| BOLD:AAX5748 | *Leptychaster anomalus* | 1 | QS | | |
| BOLD:AAJ1384 | *Leptychaster pacificus* | 2 | HG | | |
| **BOLD:AAC9615** | ***Lophaster furcilliger*** | **3** | **HG; HS** | | |
| **BOLD:ABZ8147** | ***Lophaster furcilliger*** | **2** | **BS** | | |
| **BOLD:ACE9809** | ***Lophaster furcilliger*** | **4** | **HG; QS** | | |
| BOLD:AAB9720 | *Luidia foliolata* | 12 | HG; HS | | |
| BOLD:AAB7721 | *Mediaster aequalis* | 22 | BA; HG; HS; NA | | |
| BOLD:AAC7366 | *Mesocentrotus franciscanus* | 14 | BA; NA | | |
| BOLD:AAC7305 | *Molpadia intermedia* | 10 | BA; HG; HS | | |
| BOLD:AAK8941 | *Nearchaster aciculosus* | 1 | NS | | |
| BOLD:AAD0922 | *Ophiacantha bidentata* | 9 | | BB; BE; IG; RE; SI | |
| BOLD:AAC6367 | *Ophiocten hastatum* | 4 | KS | | |
| BOLD:AAB5245 | *Ophiocten sericeum* | 22 | | BB; BE; PW; IG; RE | |
| BOLD:AAI1151 | *Ophiomusium glabrum* | 2 | VI | | |
| | **Ophiomusium lymani* | 1 | CS | | |
| BOLD:AAA9003 | *Ophiopholis aculeata* | 27 | | CH | SA |
| BOLD:AAI1097 | *Ophiopholis japonica* | 2 | HG | | |
| BOLD:AAA9002 | *Ophiopholis kennerlyi* | 22 | BA; HG; SE | | |
| BOLD:AAE1685 | *Ophiopholis* sp. *AAE1685* | 5 | BC; HG | | |
| BOLD:AAS4306 | *Ophiophthalmus cataleimmoidus* | 1 | KS | | |
| BOLD:AAF0968 | *Ophiophthalmus normani* | 3 | CS; KS | | |
| BOLD:AAD1204 | *Ophiopleura borealis* | 11 | | AG; BB; BE; LA | |
| BOLD:ACR1730 | *Ophiopus* sp. *ACR1730* | 2 | | DH | |
| BOLD:AAI1077 | *Ophioscolex corynetes* | 2 | BC; HG | | |
| BOLD:AAE1711 | *Ophiosphalma jolliense* | 5 | BC | | |
| BOLD:AAD8864 | *Ophiura luetkenii* | 15 | BA; BS; NA | | |
| BOLD:AAB2658 | *Ophiura robusta* | 25 | | BB; CH ; DI; RE | SA |
| **BOLD:AAD3481** | ***Ophiura sarsii*** | **7** | **HG** | **HB** | |
| **BOLD:ACO7183** | ***Ophiura sarsii*** | **9** | | **BB; RE** | |

| | | | | | |
|---|---|---|---|---|---|
| BOLD:AAD4189 | *Orthasterias koehleri* | 6 | BA; HG; NA | | |
| BOLD:AAI5301 | *Pannychia moseleyi* | 1 | VI | | |
| BOLD:AAD1479 | *Parastichopus californicus* | 7 | BA; HS; NA | | |
| BOLD:AAF8170 | *Pectinaster agassizi* | 3 | HG | | |
| BOLD:AAF8238 | *Pedicellaster magister* | 2 | HG | | |
| BOLD:AAD1877 | *Pentamera calcigera* | 8 | | CH ; CI; IG | |
| | **Pentamera cf. pediparva* | 2 | BA | | |
| | **Pentamera cf. pseudocalcigera* | 4 | BA; HG; HS | | |
| BOLD:AAF1583 | *Pisaster brevispinus* | 2 | NA | | |
| BOLD:AAA1290 | *Pisaster ochraceus* | 20 | BA; HG; HS; NA | | |
| BOLD:AAF0422 | *Pontaster tenuispinus* | 9 | | BB; BE | |
| BOLD:AAD7438 | *Pseudarchaster dissonus* | 6 | HG; QS | | |
| BOLD:AAH8175 | *Pseudarchaster parelii* | 1 | BS | | |
| BOLD:AAD5295 | *Pseudarchaster parelii alascensis* | 7 | HG; KS; VI | | |
| BOLD:AAE8123 | *Pseudostichopus mollis* | 2 | BS | | |
| | **Pseudostichopus tuberosus* | 2 | CS; VI | | |
| BOLD:ABZ6380 | *Psilaster andromeda* | 4 | | BB; BE | |
| BOLD:AAE8082 | *Psilaster pectinatus* | 5 | CJ; KS; QS; VI | | |
| BOLD:AAE0864 | *Psolus chitonoides* | 4 | BA; NA | | |
| BOLD:AAB7664 | *Psolus fabricii* | 16 | | CH ; RE | SA |
| BOLD:AAC9852 | *Psolus phantapus* | 7 | | IG; RE | SA |
| BOLD:ABX5586 | *Pteraster coscinopeplus* | 1 | KS | | |
| BOLD:AAE8532 | *Pteraster jordani* | 3 | KS | | |
| **BOLD:AAC7423** | ***Pteraster militaris*** | **5** | | | **SA** |
| **BOLD:AAC7424** | ***Pteraster militaris*** | **1** | **NA** | | |
| **BOLD:ABZ2331** | ***Pteraster militaris*** | **2** | **HG; NA** | | |
| BOLD:AAH7925 | *Pteraster sp. AAH7925* | 2 | HG | | |
| BOLD:AAL2213 | *Pteraster sp. AAL2213* | 1 | | RE | |
| BOLD:AAH7926 | *Pteraster tesselatus* | 2 | BA; HG | | |
| BOLD:AAD3853 | *Pycnopodia helianthoides* | 11 | BA; HG; HS; NA | | |
| BOLD:AAD3271 | *Sagenaster evermanni* | 6 | BC; NS | | |
| BOLD:AAE1365 | *Solaster dawsoni* | 8 | BA; HG; NA | | |
| BOLD:AAC8811 | *Solaster endeca* | 7 | NA | RE | SA |
| BOLD:AAD3101 | *Solaster paxillatus* | 7 | CS; KS | | |

| | | | | | |
|---|---|---|---|---|---|
| BOLD:AAF4823 | *Solaster sp. AAF4823* | 2 | NA | | |
| BOLD:AAF4824 | *Solaster sp. AAF4824* | 1 | | RE | |
| BOLD:AAD7800 | *Solaster stimpsoni* | 6 | BA; NA | | |
| BOLD:AAG3583 | *Sperosoma biseriatum* | 1 | KS | | |
| BOLD:AAJ4824 | *Stegophiura carinata* | 1 | HG | | |
| BOLD:AAE1859 | *Stegophiura nodosa* | 6 | | IG; QK | |
| BOLD:AAF3251 | *Stephanasterias albula* | 1 | | DI | |
| BOLD:ABY5959 | *Strongylocentrotus droebachiensis* | 19 | BA; HG; HS; NA | | SA |
| BOLD:AAE2166 | *Strongylocentrotus fragilis* | 5 | HG | | |
| BOLD:AAA9522 | *Strongylocentrotus pallidus* | 25 | HG | BB; CH; HB; LS; RE | |
| BOLD:AAD8117 | *Strongylocentrotus purpuratus* | 6 | BA | | |
| BOLD:AAA9523 | *Strongylocentrotus sp. AAA9523* | 2 | | RE | |
| BOLD:AAC7463 | *Stylasterias forreri* | 9 | BA; NA | | |
| BOLD:AAK1794 | *Thrissacanthias penicillatus* | 1 | BC | | |
| BOLD:AAE3748 | *Thyonidium drummondii* | 5 | | | SA |
| BOLD:AAG0209 | *Urasterias lincki* | 1 | | BE | |
| BOLD:AAD3272 | *Zoroaster ophiurus* | 6 | HG; KS; VI | | |
